# A Robust and Efficient Workflow for Heart Valve Disease Detection from PCG Signals: Integrating WCNN, MFCC Optimization, and Signal Quality Evaluation

**DOI:** 10.3390/s25216562

**Published:** 2025-10-24

**Authors:** Shin-Chi Lai, Yen-Ching Chang, Ying-Hsiu Hung, Szu-Ting Wang, Yao-Feng Liang, Li-Chuan Hsu, Ming-Hwa Sheu, Chuan-Yu Chang

**Affiliations:** 1 Department of Automation Engineering, National Formosa University, Yunlin 632301, Taiwan; 2 Smart Machinery and Intelligent Manufacturing Research Center, National Formosa University, Yunlin 632301, Taiwan; 3 Department of Electronics Engineering, National Yunlin University of Science and Technology, Yunlin 64002, Taiwan; 4 Doctor’s Program of Smart Industry Technology Research and Design, National Formosa University, Yunlin 632301, Taiwan; 5 Department of Computer Science and Information Engineering, Chaoyang University of Technology, Taichung 413310, Taiwan; 6 Department of Computer Science and Information Engineering, National Yunlin University of Science and Technology, Yunlin 64002, Taiwan

**Keywords:** convolution neural network (CNN), deep learning, heart valve diseases (HVDs), mel-frequency cepstral coefficient (MFCC), phonocardiogram (PCG) signal

## Abstract

This study proposes a comprehensive and computationally efficient system for the recognition of heart valve diseases (HVDs) in phonocardiogram (PCG) signals, emphasizing an end-to-end workflow suitable for real-world deployment. The core of the system is a lightweight weighted convolutional neural network (WCNN) featuring a key weighting calculation (KWC) layer, which enhances noise robustness by adaptively weighting feature map channels based on global average pooling. The proposed system incorporates optimized feature extraction using Mel-frequency cepstral coefficients (MFCCs) guided by GradCAM, and a band energy ratio (BER) metric to assess signal quality, showing that lower BER values are associated with higher misclassification rates due to noise. Experimental results demonstrated classification accuracies of 99.6% and 90.74% on the GitHub PCG and PhysioNet/CinC Challenge 2016 databases, respectively, where the models were trained and tested independently. The proposed model achieved superior accuracy using significantly fewer parameters (312,357) and lower computational cost (4.5 M FLOPs) compared with previously published research. Compared with the model proposed by Karhade et al., the proposed model use 74.9% fewer parameters and 99.3% fewer FLOPs. Furthermore, the proposed model was implemented on a Raspberry Pi, achieving real-time HVDs detection with a detection time of only 1.87 ms for a 1.4 s signal.

## 1. Introduction

**Figure 1:**
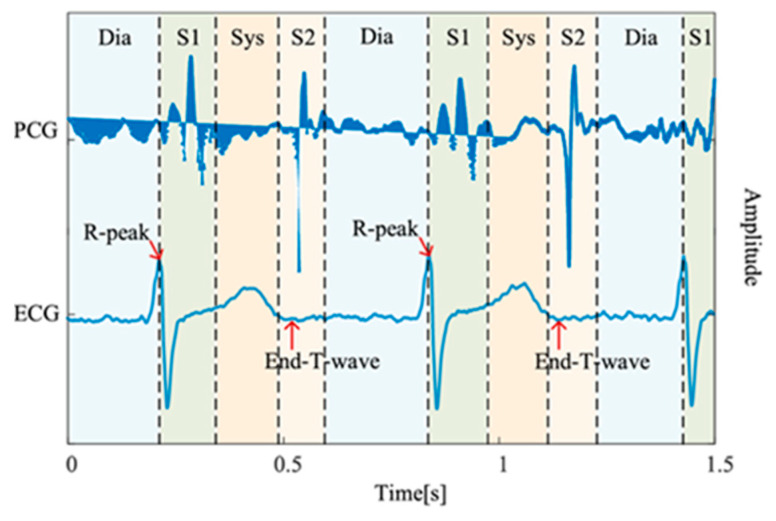
PCG recording and the four states of the PCG recording: S1, systole (Sys), and S2, diastole (Dia) [6].

The mortality rate of cardiovascular diseases (CVDs), such as heart valve diseases (HVDs), is high. The human heart has four valves that regulate the flow of blood into and out of the right and left ventricles and throughout the body: the mitral, aortic, tricuspid, and pulmonary valves. Healthy valves facilitate the body’s metabolism by preventing the backflow of blood, transporting oxygen in the blood, and removing waste products from the blood. HVDs are mainly characterized by valve damage or lesions and have a higher mortality rate relative to other CVDs [1]. HVDs can result in various conditions, such as aortic stenosis, mitral regurgitation, mitral stenosis, and mitral valve prolapse, which occur during the heartbeat cycle because of improper valve function. In severe cases, HVDs can cause arrhythmias, blood clots, ischemic strokes, heart failure, and even sudden death. The World Health Organization estimates that CVDs account for the deaths of approximately 17.9 M people worldwide each year [2]. Therefore, the early detection of CVDs, especially HVDs, is crucial. Heart sound results from the shock wave generated when blood flows through the heart during circulation [3]. This sound can be regarded as the vibration produced by a heart valve during its opening and closing. Heart valves regulate the forward flow of blood and prevent its backward flow [4]. In healthy adults, the first and second heart sounds can be clearly heard during systole and diastole, respectively. In addition to normal heart sounds, arrhythmic sounds such as heart murmurs may occur. The third heart sound is mostly heard in patients or some healthy children and adolescents, whereas the fourth heart sound is heard in some healthy older adults. Heart sounds can be heard at specific locations on the chest wall by using a stethoscope. Moreover, the amplitude of heart sounds can be recorded in a phonocardiogram (PCG). Heart sounds and PCG signals can help doctors in diagnosing HVDs; however, the process of using a stethoscope to identify heart sounds relies heavily on the clinical experience of the physician. In fact, previous studies have demonstrated that even trained clinicians may achieve only moderate inter-observer agreement when interpreting systolic murmurs [5]. Moreover, PCG signals often contain subtle acoustic features that are difficult to discern through human hearing alone, particularly in noisy environments or during the early stages of cardiovascular disease. Each complete cycle of a PCG signal typically includes two main components, the S1 and S2, corresponding to the beginning of ventricular systole and diastole, respectively. As illustrated in Figure 1 [6], S1 and S2 appear as distinct peaks in the PCG waveform. According to clinical guidelines, a resting adult heart rate ranges from 60 to 100 beats per minute [7], suggesting that a 2 second PCG recording should contain approximately 2–3 distinguishable S1–S2 cycles. The absence of such periodic structures typically indicates poor signal quality, often due to substantial noise contamination.

Recent advances in digital auscultation and deep learning techniques have shown great potential in addressing these limitations by enabling automated PCG analysis, which can effectively extract clinically meaningful patterns from either raw waveforms or time–frequency representations [5,6]. Common time–frequency feature extraction techniques include spectrograms [8,9], mel-frequency cepstral coefficients (MFCCs) [10,11,12], and wavelet transforms [10,13,14]. In the last decade, many studies have explored the identification of HVDs by using PCG signals. Machine learning [8,10,15,16,17,18,19,20], and deep learning [9,11,12,13,14,21,22,23,24,25,26,27,28,29,30], methods have been used to classify PCG signals. Deep learning models that have been employed in PCG classification include deep neural network (DNN) [11] models, AlexNet [22], convolution neural network (CNN) models [21,23,24,28,29], WaveNet [25], long short-term memory (LSTM) [26,29] models, CNN–LSTM models [12,27], and the transform network model [30]. For example, Arslan et al. [11] decomposed the PCG signal into six modes, ranging from high- to low-frequency eigenmode functions (IMF), using the Hilbert–Huang transform. They used MFCC to extract features from each eigenmode to evaluate their effect on PCG signal classification. Moreover, Arslan et al. [11] employed a genetic algorithm [31] for feature selection and utilized various machine learning methods, such as k-nearest neighbor, multilayer perceptron, support vector machine (SVM) and DNN, for PCG classification. The classification results indicated that among these machine learning methods, DNN was the most accurate (98.9%), followed by SVM (96.2%). Karhade et al. [23] proposed a time–frequency-domain deep learning (TFDDL) framework for the automatic detection of HVDs by using PCG signals. In their framework, PCG signals are denoised and then processed using the time-domain polynomial chirp transform (TDPCT) and frequency-domain polynomial chirp transform (FDPCT) to create time–frequency images of these signals. Subsequently, they employed a deep CNN model to detect HVDs from these images. Their experimental results indicated that the use of the TDPCT and FDPCT resulted in overall signal classification accuracies of 99% and 99.48%, respectively, for the GitHub PCG database [10]. However, the TFDDL framework achieved an accuracy of 85.16% in classifying PCG signals from the PhysioNet/CinC Challenge 2016 database, which indicated the framework’s susceptibility to noise. In [25], a deep WaveNet model consisting of six residual blocks with gated activation was proposed for the automated classification of five classes of PCG signals. The residual and skip connection blocks in this model accelerate convergence and prevent vanishing gradients. The aforementioned model achieved its highest accuracy of 98.20% for the normal class of PCG signals. The WaveNet model can be efficiently trained on large quantities of audio data per second but is computationally expensive. Shuvo et al. [26] proposed CardioXNet, which is a lightweight end-to-end convolution recurrent neural network model, for the automatic detection of five classes of HVDs. This model conducts representation learning and sequence residual learning. The representation learning framework of CardioXNet consists of three parallel components: a frequency feature extractor, a pattern extractor, and an adaptive feature enhancer. Its sequence residual learning framework also contains two bidirectional LSTM layers. The aforementioned model achieved an average accuracy of 99.60% in a study by Shuvo et al. However, it has numerous parameters and thus requires considerable memory storage space. In [12], MFCC was used to extract features from PCG signals, and three deep learning models were proposed for signal classification: a CNN model, an LSTM model, and the combination of a 1D CNN and LSTM network. The CNN, LSTM, and 1D CNN–LSTM models achieved accuracies of 99.1%, 98.2%, and 99.4%, respectively, and are computationally inexpensive. However, the authors of [12] excluded audio files with noise or low amplitude from their experimental database. The aforementioned model appears to exhibit low robustness to noise. Wang et al. [30] introduced PCTMF-Net, a hybrid architecture combining two parallel CNN branches with a multi-head Transformer encoder. The model used a digital Butterworth filter in preprocessing and second-order spectral analysis instead of conventional spectrograms, achieving 99.36% accuracy on the GitHub PCG dataset and 93% on PhysioNet/CinC Challenge 2016 database. Despite its accuracy and robustness to noise, the model’s complexity—2.56 M parameters—may hinder deployment on resource-constrained devices. In general, machine learning approaches rely heavily on handcrafted features and domain expertise, which may limit their generalizability. In contrast, deep learning methods can automatically learn hierarchical feature representations from data, enabling higher accuracy and better adaptability, albeit at the cost of increased computational complexity and longer training times [32].

To address the challenges of computational cost, noise robustness, and real-world deployability in HVD recognition, this study proposes a lightweight and integrated system that combines signal preprocessing, MFCC-based feature extraction, optimized CNN classification, and post hoc analysis. To facilitate comparison with previous studies, this work employs two commonly used PCG databases, which are trained independently. Specifically, the GitHub PCG database [10] is a five-class dataset, whereas the PhysioNet/CinC Challenge 2016 database [33] is a binary classification dataset. The system performs signal segmentation and preprocessing, followed by the extraction of MFCCs that capture perceptually meaningful PCG features. These features are optimized using GradCAM to guide the selection of informative dimensions, reducing input redundancy while preserving critical diagnostic cues. The classification module adopts a weighted convolutional neural network (WCNN) with a key weighting calculation (KWC) layer that adaptively emphasizes relevant channels via global average pooling, improving noise robustness. After classification, band error rate (BER) is used to evaluate signal quality and analyze the relationship between noise and misclassification. Through this integrated, low-computation workflow, the proposed system achieves high classification performance with real-time applicability on resource-constrained devices such as wearables and embedded systems. To overcome the limitations of conventional classification models that primarily focus on CNN depth or complexity, this study adopts a system-level design integrating PCG signal preprocessing, interpretable feature extraction, noise-resilient classification, and real-time deployment. This comprehensive workflow is developed not only to enhance classification accuracy but also to ensure feasibility for low-power, embedded applications. Compared with architectures proposed in previous studies, the proposed system exhibits the following novel characteristics. By integrating interpretable feature extraction, noise-resilient classification, and efficient deployment, the proposed workflow ensures both accuracy and feasibility for practical applications.

- The proposed system integrates all functional modules—including signal preprocessing, MFCC-based feature extraction, lightweight WCNN-based classification, and real-time deployment—into a unified and practical workflow for heart valve disease recognition;
- It uses MFCCs to extract the acoustic features of PCG signals and applies GradCAM-based model visualization to optimize the dimensionality of the input feature map, thereby reducing data complexity while preserving relevant signal characteristics;
- The WCNN used in the proposed architecture considerably increases this architecture’s recognition accuracy;
- The introduction of BER enables quantitative signal quality analysis, supports noise-robust modeling, and reinforces classification interpretability;
- This complete system not only demonstrates high classification accuracy, but also maintains a low-computation, lightweight, and robust design suitable for real-time execution on resource-constrained devices.

## 2. Proposed Method

**Figure 2:**
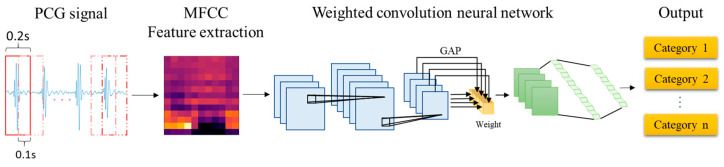
Proposed architecture for the automatic detection of HVDs.

The proposed architecture for the automatic detection of HVDs from PCG signals is illustrated in Figure 2. The feature extraction block in this architecture utilizes the MFCC technique to extract acoustic features from raw PCG data. Among the various dynamic features derived from time–frequency representations, MFCC contours have demonstrated superior overall performance in cardiac murmur classification, outperforming alternative features such as instantaneous frequency and equivalent bandwidth [34]. These MFCC features are then used as inputs for the proposed WCNN. Multiple convolution layer capture detailed features of the acoustic feature map, following which weight calculation is conducted to apply weighting to the feature map. This approach enables important features to be emphasized. Finally, a fully connected layer flattens the processed signal into a 1D signal and outputs classification results for HVD identification.

### 2.1. Feature Extraction

**Figure 3:**
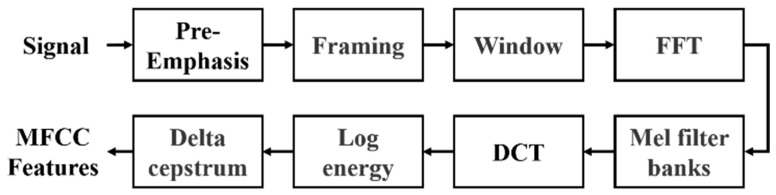
Flowchart for the calculation of MFCC features.

The proposed architecture employs MFCC, a widely used method in speech signal processing, to extract meaningful acoustic features from PCG data by simulating the human auditory system’s non-linear sensitivity to frequencies. The process involves pre-emphasis, framing, windowing, fast Fourier transform (FFT), Mel filter banks, and discrete cosine transform (DCT). The ultimate output is the MFCC features, illustrated in Figure 3. In conventional audio signal processing practice, MFCCs are typically represented as a 39-dimensional feature vector, which consists of 13 static coefficients that capture the spectral envelope of the signal, as well as 13 delta coefficients and 13 delta–delta coefficients that describe first- and second-order temporal derivatives. This representation has been widely adopted in speech analysis.

The input signals, such as those from the GitHub PCG database (8000 Hz) or PhysioNet/CinC Challenge database (2000 Hz), first undergo pre-emphasis using a high-pass filter to enhance high-frequency components. This step eliminates the influence of the vocal folds and lips during sound production and highlight the high-frequency signals obscured by the vocal system. Signals are then segmented into 0.2-second overlapping frames (1600 samples for 8000 Hz and 400 samples for 2000 Hz), with a 50% overlap. Each frame is smoothed using a Hamming window to minimize spectral leakage. The windowed frames are transformed to the frequency domain using FFT, producing spectra that are passed through 32 triangular Mel filter banks. These filters mimic human auditory sensitivity by densely spacing filters in the lower frequencies and sparsely in the higher ones. Logarithmic compression is applied to the Mel-scaled spectra, which are then transformed via DCT to derive the MFCCs. Finally, dynamic characteristics are calculated from the delta coefficient and delta–delta coefficient, reflecting the temporal variation in these coefficients. The resulting MFCC feature matrix represents the extracted acoustic characteristics of the PCG signal. Each row corresponds to a specific MFCC feature, and each column represents a frame. This MFCC matrix serves as the input to the neural network for classification tasks.

### 2.2. Parameter Settings

CNNs have been widely used in image and speech recognition applications, providing impressive results. However, CNNs behave similar to black boxes that extract and learn features automatically when provided input signals. Therefore, the effectiveness of a CNN model depends solely on its output. Improving a CNN model without having a deep understanding of its fundamentals is difficult. The reliability of the outputs of the WCNN in the proposed architecture is assessed using the Grad-CAM algorithm [35]. This algorithm calculates the gradient through backpropagation and then uses the calculated gradient as the weight of the feature map. Subsequently, it creates a heatmap representing the evidence identified within the WCNN, which illustrates this model’s focus on specific regions.

**Figure 4:**
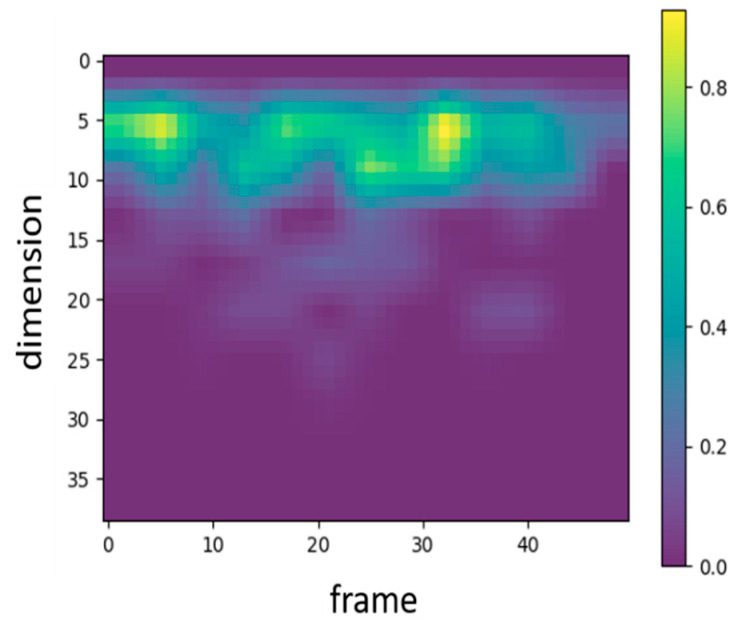
Heatmap representing the MFCC features that the proposed WCNN focuses on.

In audio signal processing, MFCC features are traditionally represented by 39 coefficients, including static, delta, and delta–delta coefficients. To examine which types of features the proposed WCNN model attends to, Grad-CAM was applied to visualize the 39-dimensional MFCC inputs. Figure 4 presents the corresponding heatmap, where the color intensity ranges from 0 to 1 and reflects the relative activation magnitude of neurons within the WCNN model. The vertical and horizontal axes denote feature dimensions and frames, respectively. As shown in Figure 4, the model primarily focuses on the static MFCCs (0–12), while paying comparatively little attention to the dynamic coefficients. Consequently, this study adopts static MFCCs as the input features in order to emphasize the most informative features and avoid potential interference from less relevant ones. In addition, to examine the influence of static feature dimensionality, the number of static MFCCs was considered to vary between 13 and 20. The final configuration employs 17 static MFCCs per frame (including the energy coefficient) and 13 frames per segment. The rationale for selecting this configuration is further detailed in Section 4.1.

To configure the input features for the proposed model, both the number of MFCC feature dimensions and the number of feature frames were parameterized. The relationship between the number of frames and the sampling rate can be expressed as follows:

(1) $$Frames=\frac{fs\times \sec-win_{length}}{win_{step}}+1,$$

where Frames represents the number of frames, fs denotes the sampling rate, sec is the signal length in seconds, $win_{length}$ denotes the samples of window length, and $win_{step}$ represents the samples of step size for each window movement. For example, when the sampling rate is 8000 Hz, the frame size is 0.2 s, and the frame overlap is 0.1 s, the window length and window step are equal to 1600 and 800 samples, respectively. Hence, a 1.4 s PCG signal comprises 13 frames. If the duration of an audio file exceeds the desired duration, the excess data are truncated; conversely, if the duration of an audio file is shorter than the desired duration, the file is zero-padded to achieve the required duration.

### 2.3. Weighted CNN Process

In general, a CNN model consists of several hidden layers, including a convolution layer, pooling layer, and fully connected layer. This model offers two advantages over DNN models: it enables weight sharing and provides location information. Weight sharing refers to sliding the same kernel across the entire image for convolution operations. In images, adjacent pixels are somewhat correlated; thus, if all images are represented in one dimension, spatial information between features might be lost. Therefore, CNN models typically have better image recognition capability than DNN models.

The main difference between the proposed WCNN model and a traditional CNN is that the proposed model includes a KWC layer. Global average pooling (GAP) is often used to replace a fully connected layer; whereas, in this layer, it is employed to calculate weights. In GAP, the feature map from each channel is replaced by its average value. This method has shown excellent performance across various domains. However, replacing a feature map with only a value leads to the loss of many features. To address this problem, the present study considered the output of GAP as the weight to emphasize feature maps with higher averages and de-emphasize those with lower averages.

**Figure 5:**
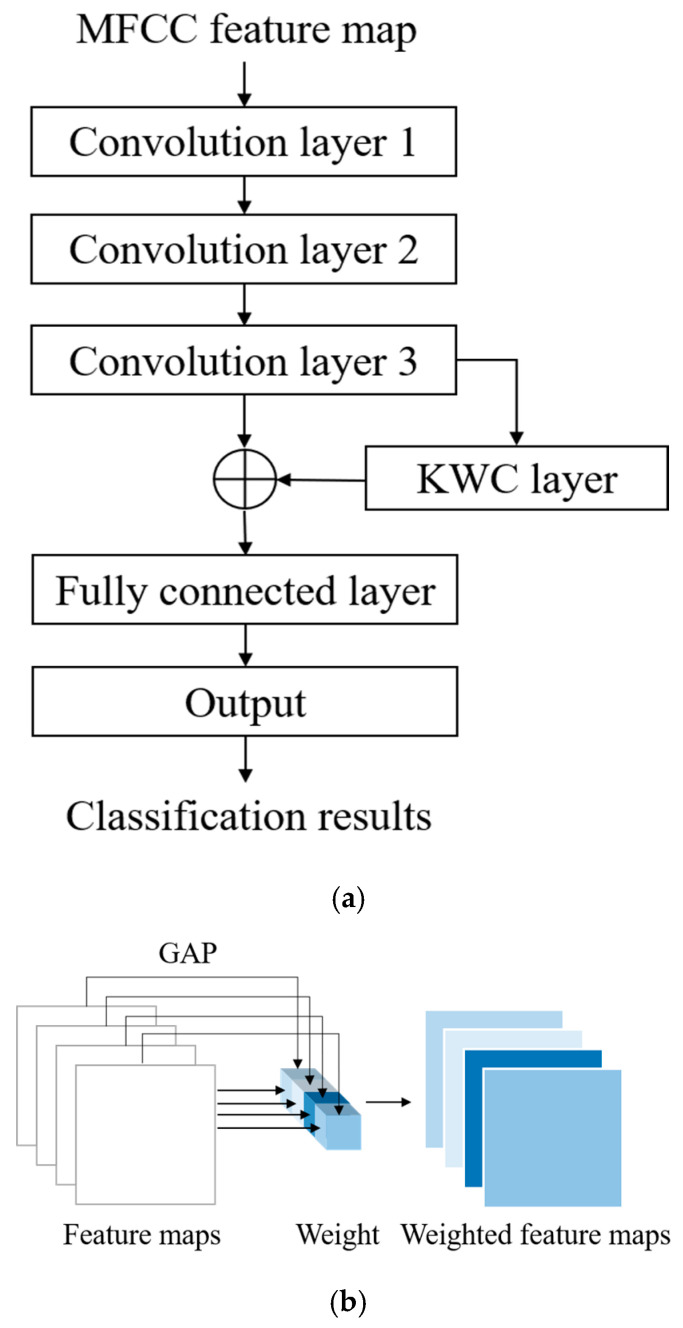
Architecture of the proposed WCNN model: (**a**) flowchart; (**b**) KWC layer of the WCNN model.

The architecture of the proposed WCNN is displayed in Figure 5. As shown in Figure 5a, the inputs of this model are MFCC feature maps, which are generated by the feature extraction block of the proposed classification architecture. The convolution layers of the WCNN enhance the details and spatial correlation of audio feature images. Moreover, the stride size can be optimized to reduce the dimensionality of the data. The outputs of the convolution layers are fed to the KWC layer, which calculates the average value for each feature map channel and treats it as the weight for the channel. The weighting process highlights the difference between important and unimportant features, allowing the model to focus on channels with higher information content. The adaptive weighting mechanism of the KWC layer also contributes strong noise robustness. Channels that consistently respond to meaningful heart sound structures tend to exhibit higher average activations, while those dominated by background noise or irrelevant fluctuations yield lower activation values. By assigning weights proportional to these average values, the KWC layer effectively downweights noisy or less informative channels and amplifies the contribution of those carrying clinically relevant features. The generated feature maps are weighted by multiplying them with their respective channel weights, as illustrated in Figure 5b. Finally, the signal is flattened by a fully connected layer, and the output layer provides the final prediction result. In each iteration, the loss between the obtained result and the target is computed. Subsequently, through backpropagation, gradients are calculated, and weights are updated to minimize the loss. This iterative training process facilitates automatic feature learning for HVD recognition.

To enhance the model’s ability to focus on informative spectral patterns and suppress irrelevant activations, the proposed WCNN integrates a KWC layer following the convolutional feature extraction. The KWC layer adaptively adjusts the significance of each channel by computing channel-wise weights via global average pooling. Specifically, for a given feature map F with dimensions C×H×W, the weight $w_{c}$ for the c-th channel is computed as:

(2) $$w_{c}=\frac{1}{H\times W}\sum_{i=1}^{H}\sum_{j=1}^{W}F_{c}(i,j),$$

where $F_{c}(i,j)$ represents the activation at location (i,j) in the c-th channel, H and W denote the height and width of the feature map, respectively. The resulting weight $w_{c}$ is then applied to rescale each channel via element-wise multiplication:

(3) $$F_{c}^{\prime }=w_{c}\cdot F_{c},$$

where $F_{c}^{\prime }$ denotes the weighted c-th channel, emphasizing more discriminative features and reducing the influence of noise-dominated channels. This process strengthens the representation of relevant heart sound components, particularly under noisy signal conditions.

The overall computational process of the WCNN integrated with the KWC layer is summarized in Table 1. The KWC layer offers a lightweight yet effective strategy for suppressing noise and directing learning capacity toward diagnostically relevant patterns in PCG signals. This mechanism contributes to the overall robustness and computational efficiency of the proposed classification system.

Detailed information on the layers of the proposed WCNN is presented in Table 2. The model receives a single-channel feature map of size 17 × 13 × 1 as input, where each segment consists of 17 static MFCCs per frame and 13 frames per segment. The first convolution layer consists of 32 kernels with a size of 2 × 2 and a stride of 1. The second convolution layer comprises 64 kernels with a size of 2 × 2 and a stride of 1. The final convolution layer contains 64 kernels with a size of 2 × 2 and a stride of 2 to reduce the data quantity by half. A batch normalization layer is present after each convolution layer. The KWC layer calculates the weight of each channel through GAP. After weighting is completed, the output feature map is flattened and fed to the fully connected layer with 128 neurons. The rectified linear unit (ReLU) activation function is used in all convolution layers and the fully connected layer, whereas the softmax activation function is used in the output layer to classify the results into five categories. The dropout function is employed to prevent overfitting.

### 2.4. Experimental Design

The hyperparameter settings for the proposed WCNN-based classification model are listed in Table 3. The number of epochs was set to 100 for both databases to ensure model convergence while avoiding unnecessary computation. The Adam optimizer was selected for its adaptive learning efficiency and stability, which make it particularly effective for training models on audio- and speech-related data. The learning rate was initialized at 0.001, which is a widely accepted baseline value for the Adam optimizer, and was adjusted through an exponential decay strategy with a decay rate of 0.95 per 10,000 iterations. This configuration ensured efficient and stable training without causing oscillation or premature convergence. Moreover, the GitHub PCG and PhysioNet/CinC Challenge 2016 databases were trained and evaluated independently, with each dataset having its own optimized batch size to accelerate training—16 for the GitHub PCG database and 128 for the PhysioNet/CinC Challenge 2016 database.

In addition, Table 3 summarizes the hyperparameters used for the different datasets, where the differences lie in the batch size and loss function. Specifically, for the GitHub PCG dataset, a relatively smaller batch size was adopted due to its limited data volume. For the PhysioNet/CinC Challenge 2016 dataset, since the task involved two classes, the loss function was chosen as binary cross-entropy, whereas categorical cross-entropy was applied for the five-class GitHub PCG dataset.

Two validation methods were employed, namely holdout validation [36] and k-fold cross-validation [36]. In holdout validation, the collected data are divided into training and testing sets, with the training set not intersecting with and typically being larger than the testing set. In the present study, holdout validation was conducted by using 90% and 10% of each dataset for training and testing, respectively. This method enabled the assessment of the model’s generalization ability and provided insights into potential overfitting problems.

In k-fold cross-validation, the collected data are divided into k equal parts, with (k–1) parts used for model training and the remaining one part used for testing in each iteration. In this study, k was set to 10. The process is repeated until each part has been used once as the test set; thus, it is performed ten times, and the average results from all iterations are used to evaluate model performance. This method enables performance assessments across different data subsets, thereby enhancing the reliability of the model’s overall evaluation. Moreover, employing 10-fold cross-validation reduces the randomness introduced by a single data split and helps confirm the model’s predictive capability for unseen data, improving its robustness and generalization. Each iteration of 10-fold cross-validation essentially represents a holdout validation process, with multiple splits and averaged outcomes to strengthen result stability. Therefore, these two validation strategies can coexist and complement each other. This design aims to verify the influence of feature settings on the proposed model’s performance while ensuring the reliability and stability of the results.

Table 4 presents the configurations of the hardware and software platforms used in this study. The hardware platform used in this study comprised an AMD Ryzen 7 5700X computer processing unit (CPU), an NVIDIA GeForce RTX 2060 SUPER graphics processing unit (GPU), and 32 GB of DDR4 RAM. The Windows 10 Pro operating system, Python version 3.8.18, and TensorFlow version 2.10.1 were installed on the software platform used in this study. Finally, the operating system for Raspberry Pi 4 was Linux 6.1.25-v8+.

### 2.5. Model Evaluation Metrics

The confusion matrix is commonly used to indicate the numbers of correctly classified and incorrectly classified samples in each category. This matrix is used to examine if a classification model is biased toward certain categories. The metrics of accuracy, precision, recall, f1-score, and kappa can be obtained from the confusion matrix and are defined in (4)–(8), respectively. Kappa is used to evaluate the performance of multi-class classification model. In (4)–(7), TP (true positives) refers to positive signals correctly identified as positive, and TN (true negatives) refers to negative signals correctly identified as negative. Conversely, FP (false positives) denotes negative signals incorrectly classified as positive, while FN (false negatives) denotes positive signals incorrectly classified as negative. In (8), $p_{o}$ is the proportion of observed counts to total counts and $p_{e}$ is the proportion of expected counts to total counts. 

In binary classification, one class must be designated as the positive class, and this choice directly affects the interpretation of the evaluation metrics such as precision, recall, and f1-score. For example, if abnormal signals are defined as the positive class, the performance metrics describe the model’s ability to correctly detect pathological conditions. Conversely, if normal signals are defined as the positive class, the same metrics reflect the model’s ability to identify non-pathological conditions. This distinction becomes particularly important in imbalanced datasets, where one class dominates the other. In this work, the abnormal (pathological) class is treated as the positive class. Precision represents the proportion of correctly predicted positive samples among all samples predicted as positive, indicating the reliability of positive predictions. Recall measures the proportion of correctly detected positive samples among all actual positives, reflecting the model’s sensitivity. The f1-score is the harmonic mean of precision and recall, balancing the trade-off between these two measures. For multi-class classification, these metrics are typically computed for each class by considering one class as positive and all others as negative, and then averaged across classes using either the macro or weighted averaging method. This allows a comprehensive evaluation of the model’s performance across all categories. For multi-class classification, these metrics are typically computed for each class by considering one class as positive and all others as negative, and then averaged across classes using either the macro or weighted averaging method. This allows a comprehensive evaluation of the model’s performance across all categories. Accordingly, the true positive rate (TPR) and false positive rate (FPR) are subsequently defined in Equations (9) and (10), respectively.

(4) $$accuracy=\frac{TP+TN}{TP+FP+FN+TN},$$

(5) $$precision=\frac{TP}{TP+FP},$$

(6) $$recall=\frac{TP}{TP+FN},$$

(7) $$f1\text{-}score=2\times \frac{precision\times recall}{precision+recall},$$

(8) $$kappa=\frac{p_{0}-p_{e}}{1-p_{e}},$$

(9) TPR=TP/(TP+FN),

(10) FPR=FP/(FP+TN),

In addition to traditional threshold-based metrics such as accuracy, precision, recall, f1-score, and kappa, this work incorporates area under the receiver operating characteristic curve (AUC-ROC) and average precision (AP) to provide a more comprehensive evaluation of model performance, particularly under imbalanced data conditions. AUC-ROC evaluates the model’s discriminative ability across varying decision thresholds by plotting the TPR against the FPR. AUC values closer to 1 indicate better separation between classes. Its continuous form is defined as:

(11) $$AUC\text{-}ROC=\int_{0}^{1}TPR(FPR)dFPR,$$

In practice, AUC can be approximated using the trapezoidal rule:

(12) $$AUC\text{-}ROC\approx \sum_{i=1}^{n-1}(FPR_{i+1}-FPR_{i})\cdot \frac{TPR_{i+1}+TPR_{i}}{2},$$

A metric derived from the precision–recall curve, AP emphasizes performance on the minority class by reflecting how precision changes across different levels of recall. It is defined as:

(13) $$AP=\int_{0}^{1}P(R)dR\approx \sum_{k=0}^{n}P(k)\cdot \Delta R(k),$$

where P(k) is the precision at the k-th recall level, ΔR(k) is the change in recall at the k-th point. In binary classification, AP directly reflects detection effectiveness for the minority class, while in multi-class classification, mean AP (mAP) is computed as the average of AP scores across all classes. It is defined as:

(14) $$mAP=\frac{1}{N}\sum_{i=1}^{N}AP_{i},$$

where $AP_{i}$ is the AP for class i, and N is the total number of classes. The use of AUC-ROC and AP is supported by Jeni et al. [37], who demonstrated that common metrics such as accuracy and f1-score may yield misleading results under class imbalance. In contrast, AUC-ROC offers greater robustness to skewed distributions, while AP is more sensitive to minority class performance and can reveal issues that may be overlooked by ROC-based evaluation.

Computational complexity and parameter count are key indicators for evaluating model efficiency. In this work, two standard metrics are adopted: FLOPs and parameter count. FLOPs measure the computational load during forward propagation, while parameter count reflects model size and storage requirements. In CNNs, convolution and fully connected layers dominate both FLOPs and parameters, whereas activation, pooling, and normalization layers contribute negligibly and are thus excluded. The numbers of FLOPs for the convolution layers and fully connected layers [38] are calculated as follows:

(15) $$FLOPs_{Conv}=2\times K_{h}\times K_{w}\times C_{in}\times H_{out}\times W_{out}\times C_{out},$$

(16) $$FLOPs_{FC}=2\times input\times output,$$

where $K_{h}$ and $K_{w}$ are the height and width of the kernel, respectively; $C_{in}$ and $C_{out}$ are the numbers of input and output channels, respectively; $H_{out}$ and $W_{out}$ are the height and width of the output feature map, respectively; and input and output represent the input and output of the fully connected layer, respectively. The numbers of parameters for the convolution layers and fully connected layers are determined using the following equations:

(17) $$Parameters_{Conv}=\left(K_{h}\times K_{w}\times C_{in}+1\right)\times C_{out},$$

(18) $$Parameters_{FC}=\left(input+1\right)\times output,$$

To systematically investigate the impact of noise on feature extraction and classification performance, a quantitative signal quality analysis framework was developed in this study. Central to this framework is the introduction of the BER, a spectral-domain metric designed to evaluate the signal-to-noise characteristics of PCG recordings. Based on existing clinical literature [39,40], the primary energy of the first (S1) and second (S2) heart sounds typically lies within 20–200 Hz, while pathological murmurs may extend up to 500–700 Hz [41,42,43]. Therefore, the 20–700 Hz band was defined as the effective signal band, and the ranges below 20 Hz and above 700 Hz were designated as noise bands. The BER is computed as the ratio of spectral energy in the effective band to that in the noise bands. The mathematical definition of BER is as follows:

(19) $$BER=\frac{\sum_{f=20}^{700}|X(f){|}^{2}}{\sum_{f<20\cup f>700}|X(f){|}^{2}},$$

where X(f) represents the power spectral density at frequency f; the numerator sums energy in the 20–700 Hz band, and the denominator sums energy outside this band. A higher BER indicates that more signal energy is concentrated within the desired heart sound frequency band (20–700 Hz), suggesting greater presence of diagnostically relevant information, whereas a lower BER reflects increased energy outside this range, often due to noise or weak cardiac activity.

## 3. Database Descriptions

### 3.1. Overview of the PCG Databases

**Figure 6:**
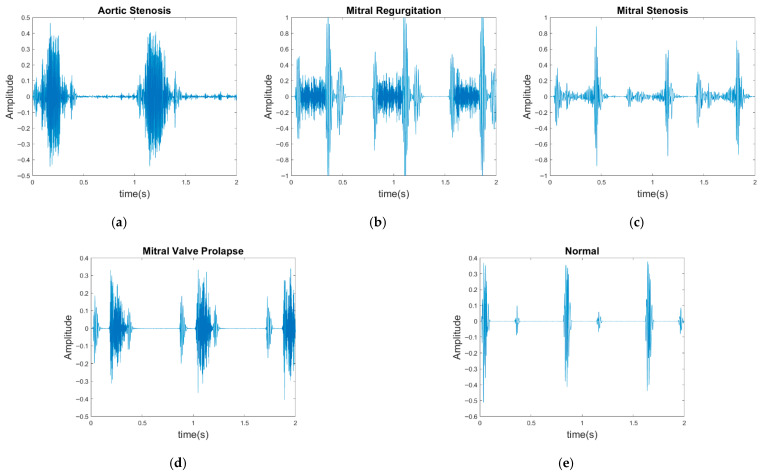
Five categories of PCG signals in the GitHub PCG database: (**a**) aortic stenosis; (**b**) mitral regurgitation; (**c**) mitral stenosis; (**d**) mitral valve prolapse; (**e**) normal.

In contrast to previous studies that have solely relied on a single dataset for evaluation, two publicly available databases were used for model evaluation in the present study: the GitHub PCG database [10] and PhysioNet/CinC Challenge 2016 database [33]. This approach enhanced the diversity of the conducted experiments and the generalizability of the results obtained. The GitHub PCG database was compiled by Yaseen et al. [10] from books and websites. During dataset construction, audio files containing excessive background noise or signal distortion were explicitly excluded to ensure high-quality recordings suitable for heart sound analysis. All retained signals were resampled to 8000 Hz to standardize frequency resolution. The database contains 1000 PCG recordings, which are classified into five categories [Figure 6a–e]: aortic stenosis, mitral regurgitation, mitral stenosis, mitral valve prolapse, and normal. Each category has its own characteristics. Each category has 200 audio files with durations ranging from approximately 1.2–4 s.

**Figure 7:**
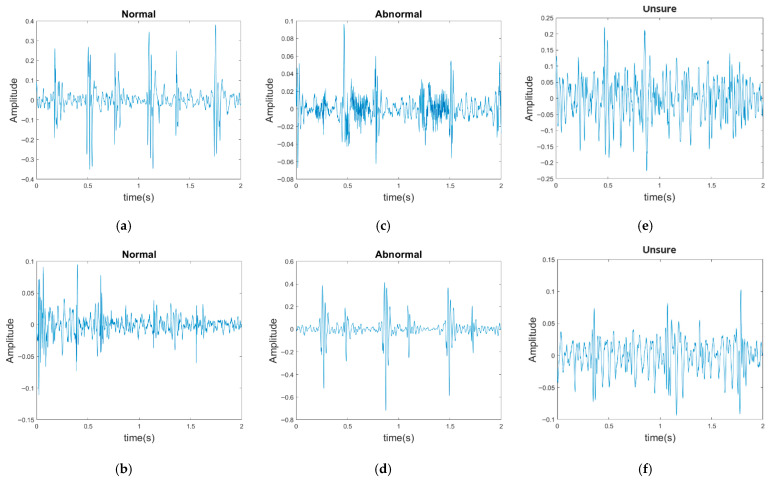
Three categories of PCG signals in the PhysioNet/CinC Challenge 2016 database: (**a**,**b**) normal; (**c**,**d**) abnormal; (**e**,**f**) unsure.

The PhysioNet/CinC Challenge 2016 database [33] comprises six folders [i.e., folders (a)–(f)] containing a total of 3240 heartbeat recordings with durations ranging from 5 to 120 s. All recordings in this database have been resampled to 2000 Hz and are available in the .wav format. The database includes heartbeat recordings of healthy and pathological patients, including children and adults. The PhysioNet/CinC recordings were obtained in uncontrolled clinical environments, as described in the official documentation. Consequently, many recordings are affected by various sources of noise, such as breathing, speech, stethoscope friction, and intestinal sounds. Heart sound signals are classified into three categories in the database: normal, abnormal, and unsure (Figure 7). Figure 7a,b depict normal PCG signals, Figure 7c,d depict abnormal PCG signals, and Figure 7e,f depict “unsure” PCG signals. PCG signals with the same label differ considerably from each other and are affected by noise. The 279 “unsure” PCG recordings in the PhysioNet/CinC Challenge 2016 database were removed, resulting in 2961 recordings remaining in the database. The class distribution of these recordings was imbalanced, with 2389 and 572 recordings (ratio of 4:1) representing normal and abnormal heart sounds, respectively.

Table 5 presents the key characteristics of the two datasets employed for model evaluation, including the number of recordings, class distribution, duration range, sampling rate, and presence of noise. The GitHub PCG database contains noise-free recordings, whereas the PhysioNet/CinC Challenge 2016 database includes recordings with varying noise levels collected in real-world clinical settings. Together, these datasets provide a balanced experimental foundation, enabling the assessment of model performance under both clean and noisy conditions.

### 3.2. Dataset Partitioning and Validation Strategy

Each PCG recording was standardized to a fixed length of 1.4 seconds (13 frames). Recordings longer than 1.4 seconds were truncated, whereas shorter recordings were zero-padded. Thus, each file corresponded to exactly one segment, ensuring no overlap or duplication. Dataset partitioning was performed strictly at the audio file level. At the beginning of the experiment, complete audio recordings from each category were randomly selected for the test set, and the remaining files were assigned to the training set. This procedure guaranteed that no identical audio file or its segments appeared in both training and testing datasets.

Two validation methods were employed: holdout validation with three different train–test splits (90–10%), and 10-fold cross-validation. For the GitHub PCG dataset, both balanced and random sampling strategies were considered within the 10-fold framework to investigate the influence of sampling design on classification performance. In the balanced sampling setting, 20 test samples were selected from each category per fold to ensure equal representation. In the random sampling setting, 100 test samples were selected per fold without restrictions on class distribution, resulting in natural variation across categories.

No additional data balancing preprocessing was applied to the PhysioNet/CinC Challenge 2016 dataset. This decision was made to preserve the original data imbalance and to evaluate the model under conditions resembling real-world applications. It also ensured consistency with most related studies, which similarly trained and tested their models on the original dataset. This design allows a fair comparison with prior work and demonstrates the robustness of the proposed model under realistic imbalanced distributions.

## 4. Analytical Results, Experimental Results and Discussion

The number of MFCC feature dimensions affects the accuracy of the model and the number of parameters required. Therefore, analysis was conducted to identify a suitable number of MFCC feature dimensions.

### 4.1. Determination of the Suitable Number of MFCC Feature Dimensions

The effects of the numbers of MFCC feature dimensions and feature frames on the accuracy of the proposed classification architecture were investigated using the GitHub PCG database and PhysioNet/CinC Challenge 2016 database (Table 6 and Table 7, respectively). The numbers of MFCC feature dimensions and feature rows were varied from 13 to 20 and from 9 to 16, respectively. Due to the consistent trend of performance across all evaluation metrics (including accuracy, precision, recall, and f1-score), and considering that accuracy is a representative metric of overall classification performance, only accuracy is reported in the main text in order to maintain brevity. Table 6 and Table 7 detail the impact of MFCC dimensions (from 13 to 20) and the number of frames (from 9 to 16) on recognition accuracy when using the GitHub database and the physioNet/CinC Challenge 2016 database, respectively.

Each accuracy value in the tables is based on both holdout validation and 10-fold cross-validation. The results presented in Table 6 indicate that the accuracy of the proposed classification architecture on the GitHub PCG database increased with the number of MFCC feature dimensions and the number of frames. The highest average accuracy for different numbers of MFCC feature dimensions (99.24%) was achieved when the number of frames was 13. Moreover, the highest average accuracy for different numbers of frames (99.3%) was achieved when the number of MFCC feature dimensions was 17. Increasing the numbers of MFCC feature dimensions and feature frames improved the classification accuracy; however, this improvement was not indefinite. The accuracy peaked to 99.6% when the numbers of dimensions and frames were 17 and 13, respectively. Thus, the optimal numbers of MFCC feature dimensions and feature frames for the GitHub PCG database were 17 and 13, respectively. 

The results presented in Table 7 suggest that the accuracy of the proposed classification architecture on the PhysioNet/CinC Challenge 2016 database increased with the number of MFCC feature dimensions and the number of frames. The highest average accuracy for different numbers of MFCC feature dimensions (90.465%) was achieved when the number of frames was 13. Moreover, the highest average accuracy for different numbers of frames (90.431%) was achieved when the number of MFCC feature dimensions was 17. The accuracy peaked to 90.74% when the numbers of dimensions and frames were 17 and 13, respectively. Thus, the optimal numbers of MFCC feature dimensions and feature frames for the PhysioNet/CinC Challenge 2016 database were 17 and 13, respectively.

The stride of convolution layers refers to the interval with which the convolutional filter moves over the input data, and this parameter directly affects the output size of the feature map. Increasing the stride reduces the size of the feature map, thereby reducing the spatial resolution of the output. However, an excessively large stride can result in the loss of useful information. Conversely, a smaller stride improves the spatial resolution, allowing the model to capture finer details. In the proposed WCNN model, the stride of the third convolution layer is set to 2, which reduces the feature map size by half. This reduction not only decreases the computational burden and memory usage but also accelerates the training process and reduces the model size. Table 8 presents the variations in the number of model parameters with the numbers of MFCC feature dimensions and frames. If the number of MFCC feature dimensions is an odd value, the last part of the input that is smaller than the stride is dropped, leading to different input sizes with the same number of parameters. The number of model parameters was 312,357 when the numbers of MFCC feature dimensions and feature frames were 17 and 13, respectively.

**Figure 8:**
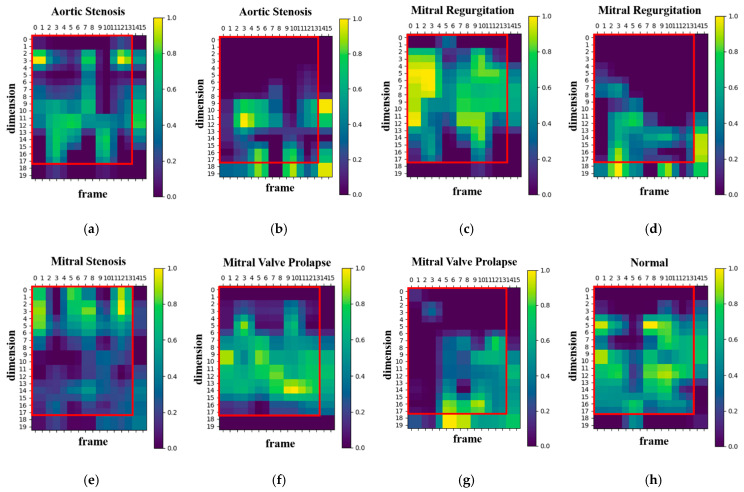
Heatmaps of the proposed model’s focus areas: (**a**) heatmap of correct predictions for the class aortic stenosis, (**b**) heatmap of incorrect predictions for the class aortic stenosis, (**c**) heatmap of correct predictions for the class mitral regurgitation, (**d**) heatmap of incorrect predictions for the class mitral regurgitation, (**e**) heatmap of correct predictions for the class mitral stenosis, (**f**) heatmap of correct predictions for the class mitral valve prolapse, (**g**) heatmap of incorrect predictions for the class mitral valve prolapse, (**h**) heatmap of correct predictions for the class normal.

To better understand the developed model’s decision-making process, the Grad-CAM algorithm [35] was used to generate a heatmap shown in Figure 8. The brighter areas in the generated heatmap indicate a higher level of attention from the model. Figure 8 illustrates the visualization results for both correct and incorrect predictions across four disease categories (aortic stenosis, mitral regurgitation, mitral stenosis, mitral valve prolapse) and normal heart sounds. Among these, mitral stenosis and normal achieved 100% prediction accuracy, so no heatmaps for incorrect predictions are included. Moreover, the vertical and horizontal axes in the heatmap represent the numbers of MFCC feature dimensions and feature frames, respectively. The red boxes in Figure 8 indicates the feature range selected in this study (17 dimensions and 13 frames). For aortic stenosis, Figure 8a and Figure 8b show the heatmaps when the predictions are correct and incorrect, respectively. Similarly, Figure 8c,d display the heatmaps for mitral regurgitation in correct and incorrect predictions. For mitral stenosis, Figure 8e illustrates the heatmap when predictions are correct, while for mitral valve prolapse, Figure 8f and Figure 8g depict the heatmaps for correct and incorrect predictions, respectively. Lastly, Figure 8h presents the heatmap for normal heart sounds during correct predictions.

The results reveal that, in cases of correct predictions, the proposed model’s attention areas are concentrated within the MFCC range selected for this study (17-dimension, 13-frame). Conversely, when predictions are incorrect, the proposed model’s attention areas deviate from this range. Increasing the numbers of MFCC feature dimensions and frames may lead to a loss of focus because of the inclusion of unnecessary features, which affects the proposed model accuracy. Therefore, selecting the appropriate feature range is crucial for enhancing the proposed model’s performance.

**Figure 9:**
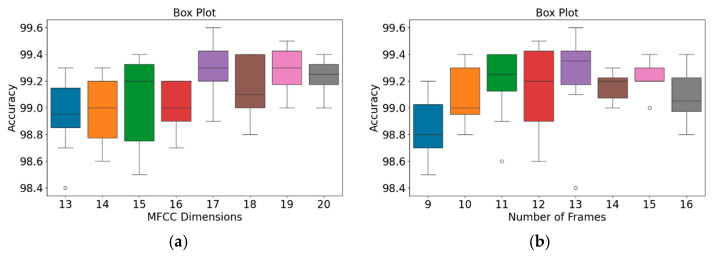
Box plots of model accuracy variations for the GitHub PCG database: (**a**) MFCC feature dimensions; (**b**) Number of frames. The central line indicates the median, the box represents the interquartile range (Q1–Q3), and the lines show the minimum and maximum values; outliers are marked as individual points.

**Figure 10:**
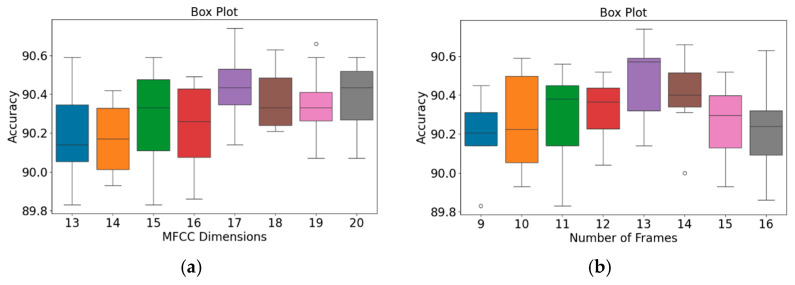
Box plots of model accuracy variations for the PhysioNet/CinC Challenge 2016 database: (**a**) MFCC feature dimensions; (**b**) Number of frames. The central line indicates the median, the box represents the interquartile range (Q1–Q3), and the lines show the minimum and maximum values; outliers are marked as individual points.

In addition, an independent analysis of the impact of MFCC dimensions and the number of frames on model accuracy was conducted. Figure 9 and Figure 10 present box plots of the variations in model accuracy with respect to the numbers of MFCC feature dimensions and feature frames for the GitHub PCG and PhysioNet/CinC Challenge 2016 databases, respectively. In these box plots, the central horizontal line denotes the median (Q2), the box edges correspond to the first (Q1) and third (Q3) quartiles, and the whiskers represent the minimum and maximum values within 1.5 times the interquartile range, while outliers are indicated by individual points. The use of box plots allows visualization of accuracy distributions obtained across multiple folds of cross-validation, thus presenting a range of values rather than a single accuracy result. This approach highlights both the central tendency and the variability of the model performance under different parameter configurations. As depicted in Figure 9a, when the number of MFCC feature dimensions was set to 17, the model achieved its highest accuracy, and the range from Q1 to Q3 was relatively narrow, indicating stable results. Moreover, as indicated in Figure 9b, when the number of feature frames was set to 13, the model achieved its highest accuracy (99.6%) and highest average accuracy. The outlier accuracy value of 98.4% in Figure 9b was caused by the number of MFCC feature dimensions being only 13. This feature dimension setting resulted in the model extracting relatively few features, which affected the stability and accuracy of its results.

The patterns presented in the PhysioNet/CinC Challenge 2016 database (Figure 10) closely resemble those observed for the GitHub PCG database (Figure 9). According to Figure 10a, when the number of MFCC feature dimensions was set to 17, the range from Q1 to Q3 was relatively narrow, and the model achieved its highest average accuracy; thus, the model’s results were relatively stable under this setting. Figure 10b indicates that when the number of frames was 13, the model achieved its highest accuracy (90.74%) and highest average accuracy; thus, this frame setting resulted in the optimal model accuracy. In summary, the highest model accuracy values for the GitHub PCG database and PhysioNet/CinC Challenge 2016 database (99.6% and 90.74%, respectively) were achieved when the numbers of MFCC feature dimensions and feature frames were set to 17 and 13, respectively, with a total of 312,357 parameters.

The variations in the accuracy and number of parameters of the WCNN-based classification model were investigated with the number of MFCC feature dimensions under the same number of frames (i.e., 13 frames). As presented in Table 9, when the number of MFCC feature dimensions was set to 13, the number of parameters was 230,437, and the model accuracy was 98.4%. To improve the model accuracy, the number of MFCC feature dimensions was increased to 17, which resulted in the model using 312,357 parameters and achieving an accuracy of 99.6%. However, when the number of MFCC feature dimensions was set to 39 (13 dimensions each for static features, delta features, and delta–delta features), the number of parameters increased to 762,917, but the accuracy slightly decreased to 99.2%. Thus, the addition of dynamic features increased the computational complexity but reduced the model accuracy.

### 4.2. Experimental Results

To determine the optimal for model training, experiments were conducted with varying batch sizes for both datasets, and the corresponding classification accuracies were evaluated, as summarized in Table 10. For the GitHub PCG database, batch sizes of 8, 16, and 32 were tested, resulting in accuracies of 99.3%, 99.6%, and 99.4%, respectively. The batch size of 16 yielded the highest accuracy and was therefore selected as the optimal configuration. For the PhysioNet/CinC Challenge 2016 database, batch sizes of 64, 128, and 256 were compared, producing accuracies of 89.62%, 90.7%, and 89.45%, respectively. The batch size of 128 achieved the best performance and was adopted for subsequent experiments. These results indicate that the optimal batch size depends on the dataset scale and complexity, with smaller batches favoring datasets of limited size and larger batches being more effective for larger, more variable datasets.

**Figure 11:**
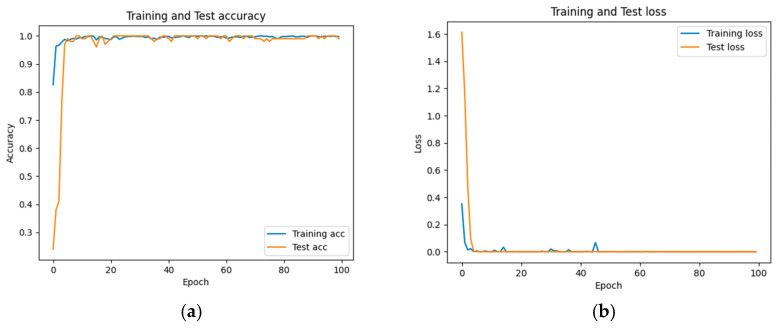
Training and testing results of the proposed model for the GitHub PCG database: (**a**) accuracy; (**b**) loss.

**Figure 12:**
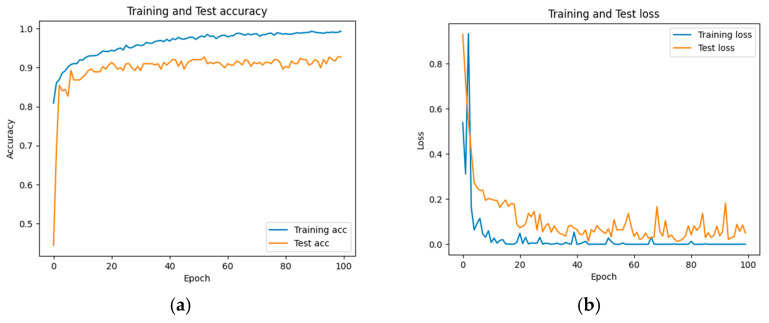
Training and testing results of the proposed model for the PhysioNet/CinC Challenge 2016 database: (**a**) accuracy; (**b**) loss.

**Figure 13:**
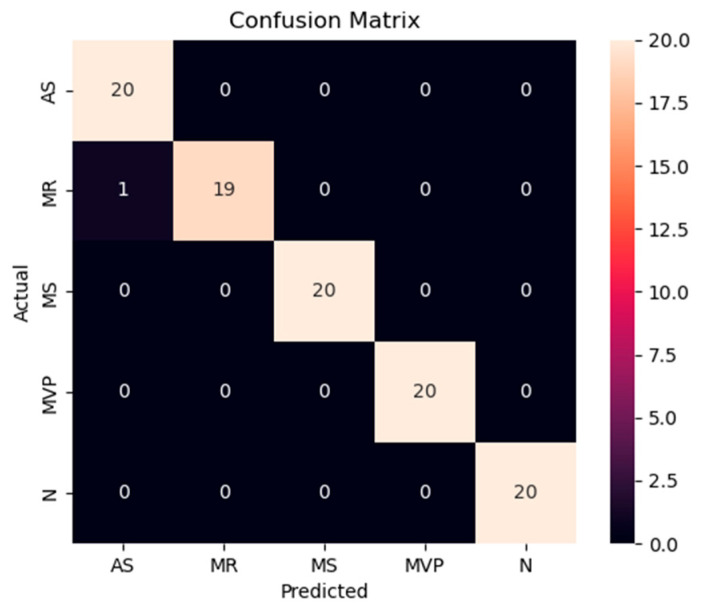
Confusion matrix of the proposed model for testing data from the GitHub PCG database.

**Figure 14:**
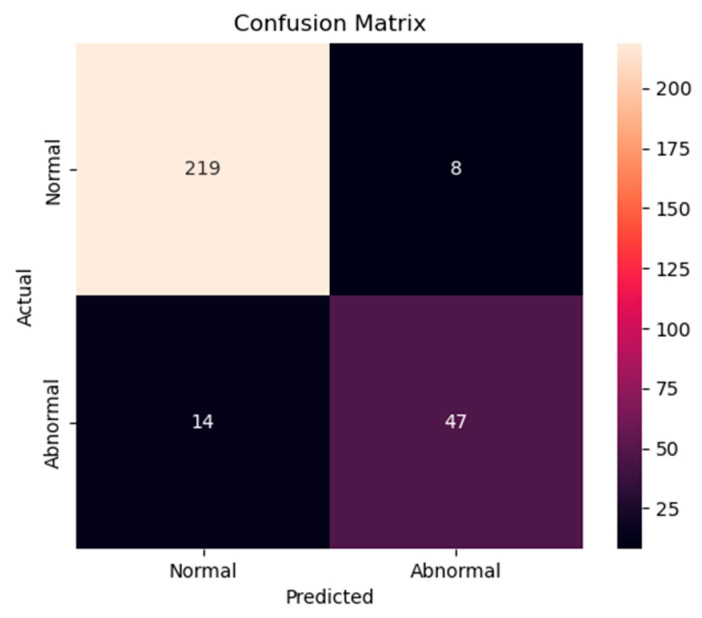
Confusion matrix of the proposed model for testing data from the PhysioNet/CinC Challenge 2016 database.

The accuracy and loss results obtained for the proposed model on the GitHub PCG database are displayed in Figure 11a and Figure 11b, respectively. Figure 11 indicates that the model converged rapidly, and its training parameters stabilized. The accuracy and loss results obtained for the proposed model on the PhysioNet/CinC Challenge 2016 database are depicted in Figure 12a and Figure 12b, respectively. Figure 12 indicates that the training accuracy increased sharply during the first 15 epochs and then gradually stabilized as the model gradually reached the optimal solution. Based on the convergence behavior observed in Figure 11 and Figure 12, the model achieved stable accuracy and loss values well before 100 epochs; therefore, 100 epochs were selected as an appropriate balance between convergence and computational efficiency. Figure 13 and Figure 14 depict the confusion matrices of the proposed model for testing data from the GitHub PCG and PhysioNet/CinC Challenge 2016 databases, respectively. The proposed model achieved accuracy values of 99% and 93.26% for the aforementioned data, respectively.

To evaluate the importance of the KWC module in enhancing noise robustness, a detailed ablation study was conducted using both clean and noisy datasets. The results are summarized in Table 11 and Table 12. As shown in Table 11, using the GitHub PCG dataset for comparison, the proposed model’s accuracy decreased from 99.6% to 98.9% after removing the KWC module, with the f1-score also dropping from 99.6% to 98.9%. Similarly, other metrics such as precision and recall have declined, with the kappa value dropping from 99.5% to 98.6%. On the PhysioNet/CinC Challenge 2016 dataset, as shown in Table 12, the removal of the KWC module caused accuracy to decrease from 90.74% to 88.37%, and the f1-score to drop significantly from 84.94% to 69.87%. Precision and recall also saw notable reductions, from 85.85% to 76.74% and 83.46% to 64.71%, respectively. It is worth noting that the addition of the KWC layer results in a more significant improvement in various metrics for the PhysioNet/CinC Challenge 2016 dataset compared to the GitHub PCG dataset.

The PhysioNet/CinC Challenge 2016 dataset is more heavily impacted by noise, and the KWC layer effectively extracts PCG features, thereby enhancing the model’s ability to capture the target signals and reducing the noise’s impact on classification performance. The inclusion or exclusion of the KWC layer does not affect the parameters because the KWC layer is used as a weight, which is ultimately multiplied with the corresponding channels of the feature map. Therefore, even with the addition of the KWC layer, it will not increase the parameters of the model. These results underscore the critical role of the KWC module in enhancing the proposed model’s performance. The experiments conducted on both datasets consistently demonstrate that this module significantly improves classification accuracy, f1-score, and other evaluation metrics. These results align with the theoretical basis that the KWC layer suppresses irrelevant or noisy information by applying adaptive weights to each feature map channel based on their global average activations.

To ensure the stability of the proposed WCNN-based classification model, a comprehensive analysis and comparison of the results from holdout validation and 10-fold cross-validation were conducted. Multiple evaluation metrics, including accuracy, precision, recall, f1-score, and kappa, were used to evaluate the proposed model’s performance from multiple perspectives. Table 13 presents the proposed model performance results in 10-fold cross-validation for the GitHub PCG database. In the 10-fold cross-validation for this database, the proposed model exhibited average accuracy, precision, recall, f1-score, and kappa values were 99.6%, 99.6%, 99.59%, 99.61%, and 99.5%, respectively.

Table 14 lists the average accuracy of the proposed model for each category in the 10-fold cross-validation with the GitHub PCG database. The overall accuracy for each class was above 99%. The highest accuracy rate of 100% was observed for the “normal” and “mitral stenosis” classes. The aforementioned results proved that the proposed model achieved stable performance and balanced training for each category on the GitHub PCG database. Table 15 presents the model performance results in 10-fold cross-validation for the PhysioNet/CinC Challenge 2016 database. For this database, the highest model accuracy rate was achieved in the ninth cross-validation, as shown in Figure 12a. In the 10-fold cross-validation for the aforementioned database, the average accuracy, precision, recall, and f1-score values were 90.74%, 85.85%, 83.46%, and 84.94%, respectively. Table 16 presents the overall accuracy of the proposed model for each category in the 10-fold cross-validation with the PhysioNet/CinC Challenge 2016 database. The accuracy rates for the normal and abnormal categories were 96.48% and 77%, respectively.

For the PhysioNet/CinC Challenge 2016 database, identifying abnormalities is more challenging than identifying normal scenarios because many PCG records are affected by noise, with considerable variations existing between PCG signals having the same label (Figure 7). In addition, the ratio of abnormal to normal heart sound data in the aforementioned database is approximately 1:4. To avoid excessive bias toward specific classes in the training set, the original class distribution was preserved across all folds during the training and testing processes in this work. By contrast, Shuvo et al. [26] achieved an overall accuracy of 86.57% on the same dataset, with class-wise accuracies of 93.27% for normal and 59.06% for abnormal. Compared with these results, the proposed model improved the recognition of abnormal heart sounds to 77%, substantially higher than the 59.06% reported in [26]. This demonstrates that, despite the data imbalance, the proposed model effectively reduces the gap between imbalanced data classes and improves the classification performance for the minority class. Thus, the classification model proposed in this paper has superior performance and stability to those of existing models in classifying the PhysioNet/CinC Challenge 2016 database.

Models trained on imbalanced datasets may exhibit bias toward the majority class due to uneven gradient accumulation during the optimization process. To mitigate this evaluation bias, AUC-ROC and AP were adopted as complementary metrics to accuracy, enabling a more comprehensive and reliable assessment of the model’s performance, particularly for minority classes. Accordingly, 10-fold cross-validation was conducted on both the GitHub PCG (balanced) and PhysioNet/CinC Challenge 2016 (imbalanced) datasets, with AUC-ROC and AP calculated as the primary evaluation indicators. As shown in Table 17 and Table 18, both AUC-ROC and AP were used. On the balanced GitHub PCG dataset, the model achieved near-perfect performance, with average AUC-ROC and AP scores of 0.9998 and 0.9992, respectively, and a negligible difference of 0.0006 between the two metrics. This result aligns with theoretical expectations, where ROC and PR curves typically exhibit similar behavior under balanced class distributions.

In contrast, the imbalanced PhysioNet/CinC 2016 dataset (approximate class ratio of 4:1) yielded an average AUC-ROC of 0.8485 and AP of 0.868, with a modest difference of 0.02, indicating that AP is more sensitive to minority class performance. Despite this gap, the consistently high AP score suggests that the model maintained stable and accurate recognition of the minority class (Abnormal) without requiring any data balancing preprocessing.

According to Mandrekar [44], AUC values between 0.8 and 0.9 are considered “excellent,” and Maxwell et al. [45] emphasized that AP values close to 1 indicate strong prediction quality, while those below 0.5 indicate poor minority class performance. The AUC-ROC of 0.8485 and AP of 0.868 achieved by the proposed model on the PhysioNet/CinC 2016 dataset fall within the “excellent” range, demonstrating that even without data balancing, the model can effectively identify minority class instances. This performance highlights the robustness of the proposed architecture and supports the use of AUC-ROC and AP as complementary and reliable metrics for real-world imbalanced classification problems.

### 4.3. Evaluation of Model Stability Across Balanced and Random Sampling Using 10-Fold Cross Validation

Based on the sampling strategies described in Section 3.2, the GitHub PCG database was evaluated under both balanced and random sampling conditions within a 10-fold cross-validation framework. The following presents the experimental results obtained under these two settings, with particular emphasis on the model’s stability and classification performance. The experimental results under balanced sampling are presented in Table 19. As shown, the confusion matrices indicate that most folds achieved error-free classification, with only a very small number of misclassifications in certain folds. The overall average accuracy reached 99.6%, demonstrating the stability of the model. Further examination of the average per-class accuracies presented in Table 14 shows values of 99.5% for AR, 99% for MR, 100% for MS, 99.5% for MVP, and 100% for N. These results indicate that, under balanced conditions, the model demonstrates exceptionally high recognition capability across all categories.

The results under random sampling are shown in Table 20 and Table 21. In this case, the overall average accuracy slightly decreased to 99.4%. While the difference is minor, the confusion matrices reveal that certain folds contained disproportionately large numbers of specific classes. For example, in Fold-2 and Fold-4 the MVP class appeared 28 times in the test set, which reduced its representation in the training set and led to a drop in classification accuracy. Table 21 reports the average per-class accuracies over all folds: 99.41% for AR, 99.61% for MR, 100% for MS, 98.33% for MVP, and 100% for N. Although the MVP class showed a slight decline compared with balanced sampling, its accuracy remained as high as 98.33%, while all other classes maintained accuracies above 99%. These findings demonstrate that even under imbalanced sampling conditions, the model retained strong classification performance and generalization capability.

These results provide important evidence for applying the proposed approach to another dataset, the PhysioNet/CinC Challenge 2016 database, where the ratio of normal to abnormal samples is approximately 4:1, creating an imbalance. No balancing techniques were applied; instead, the same model and training strategy were used, namely extracting MFCC features as input, employing the WCNN architecture with KWC layers, and training with 10-fold cross-validation using the Adam optimizer. Under these settings, the model maintained strong performance, with reliable recognition of minority classes. To address potential distortion of accuracy due to imbalance, AUC-ROC and AP metrics were additionally reported, providing a more sensitive and reliable evaluation of minority-class performance.

A comparison was also made with the Cardi-Net model proposed in [21]. According to the results shown in Figure 7 of [21], Cardi-Net achieved an overall average accuracy of 98.68% on the GitHub PCG database, with fold-wise accuracies ranging between 95.6% and 99.6%. By contrast, the proposed method achieved an overall average accuracy of 99.6% on the same dataset, with fold-wise accuracies consistently ranging from 98% to 100%, indicating smaller fluctuations. This highlights the superior stability and stronger robustness of the proposed model under imbalanced conditions.

In summary, although class distribution balance can influence model performance to some extent, the architecture and training strategy designed in this study enabled highly stable and accurate classification even under imbalanced scenarios. This robustness ensures reliable application to practical datasets such as PhysioNet, where class imbalance is present. Moreover, the use of AUC-ROC and AP further reflects the model’s capability in minority-class recognition, thereby validating the reliability and generalizability of the proposed approach.

### 4.4. Model Comparison and Discussion

The proposed lightweight framework achieved accuracy values of 99.6% and 90.74% for the GitHub PCG and PhysioNet/CinC Challenge 2016 databases, respectively. The model achieved the aforementioned performance with approximately 312.357 K parameters and approximately 4.5 M floating-point operations per second (FLOPs). To ensure fair comparison among different models, all comparative evaluations in this study were conducted using the same datasets—GitHub PCG and PhysioNet/CinC Challenge 2016. Accuracy was selected as the primary evaluation metric, representing overall classification performance, and was supplemented by parameters and FLOPs to assess architectural efficiency. Together, these metrics establish a consistent and representative benchmarking framework that ensures the comparability of results. Table 22 presents a comprehensive comparison between the proposed classification model and various relevant novel models in terms of their parameters, number of FLOPs, classifier, and accuracy. In addition, Table 23 provides a detailed summary of the data preprocessing strategies and the train/validation/test distributions used in each referenced study.

The paper [10,11,12], and this work all use MFCC as the data preprocessing method. The method in [10] combines MFCC, DWT, and SVM. It has a window size of 240, with a 33% overlap, and uses a 256-point FFT, ultimately selecting 19 MFCC feature dimensions. The feature vector length for the DWT is 24. Although the use of the DWT increases the complexity of feature extraction, it reduces the number of FFT points, which reduces the computational burden. The model proposed in [11] combines MFCC with empirical mode decomposition (EMD) and Hilbert–Huang transform. The aforementioned study does not provide specific details regarding how MFCC is used in the model; however, the use of EMD and the Hilbert–Huang transform considerably increases the complexity of preprocessing. The literature [12] combines MFCC with a 1D CNN-LSTM architecture. This model uses a window size of 2048, with 25% overlap; a 2048-point FFT; and 13 MFCC feature dimensions. This combination effectively leverages the strengths of the CNN and LSTM network, thus making the model suitable for processing complex sound data that include time series. The proposed method in this work combines MFCC with WCNN, using a window size of 1600, with 50% overlap; a 2048-point FFT; and 17 MFCC feature dimensions. Although the EMD-based MFCC processing method used in [10] improves the effectiveness of feature extraction, its high computational complexity limits its use in real-time applications. By contrast, the 19-dimensional features used in [9] provide more information; however, as presented in Table 6 and Table 7, increasing the number of feature dimensions increases the computational complexity but does not always improve the recognition performance. The model proposed in the present paper, which has a similar MFCC processing configuration to that used in [12], can achieve high accuracy while avoiding excessive computational burden. Preliminary simulations (Table 6 and Table 7) revealed that the model proposed in the present paper achieved its highest accuracy when the number of MFCC feature dimensions was 17.

This work, along with studies [23,24,28,29], adopts CNN-based architectures. Therefore, an in-depth comparison and analysis were conducted focusing on design choices such as kernel size, stride, max pooling, and dropout. The model developed in [23] uses the common two-dimensional convolution and pooling strategy, a 3 × 3 kernel size, and a stride of 1. Moreover, it employs 2 × 2 max pooling to gradually reduce the spatial dimensions of the generated feature map. The model developed in [24] performs 1D convolution operation with a stride of 2 or 3. It also conducts 2 × 2 max pooling and employs two dropout rates (0.15 and 0.30) to enhance the model’s generalization performance and noise resistance. Chen et al. [28] proposed a CNN model based on VGG16, consisted of three stages of 3 × 3 convolutions followed by max pooling layers, reducing the feature dimensions from 128 × 128 to 16 × 16. The model included seven convolutional layers and four fully connected layers, with a final softmax classifier for binary classification. Nguyen et al. [29], on the other hand, implemented a lightweight CNN with only three convolutional layers and two fully connected layers. Their model design utilized 3 × 3 convolutions with ReLU activations, batch normalization, and 2 × 2 max pooling, followed by a flattening operation leading to fully connected layers of sizes 100 and 5, with softmax output. The WCNN-based classification model developed in the present study uses 2 × 2 kernels and a stride of 1 in its first two convolution layers, which helps it to process fine input features. Instead of using max pooling to reduce feature dimensions, the proposed model adopts a stride of 2 in its third convolution layer, thereby preserving more of the original information. Moreover, this model incorporates an innovative KWC layer, which enhances the model’s abstraction and representation capabilities through the weighted computation of key features, thereby further optimizing overall model performance. Finally, the dropout function is employed to prevent overfitting in the proposed model.

Using a larger stride in convolution layers can effectively reduce the spatial dimensions of the generated feature map while enabling the capture of more complex features. Compared with convolution, max pooling is a simpler and faster method for reducing dimensions; however, it results in the loss of some detailed information. Model designers must attempt to achieve a suitable balance between retaining crucial information and improving computational efficiency. The KWC layer used in the WCNN-based model developed in this study not only increases the model’s computational efficiency but also improves its ability to recognize key features, which is critical for increasing the accuracy and efficiency of signal classification.

Computational complexity and parameter count are important criteria for evaluating model efficiency in addition to classification accuracy. For clarity, the comparison is presented in stages, beginning with studies that reported only accuracy, followed by those that also provided complexity measures such as parameters and FLOPs. As shown in Table 22, many earlier works lack full complexity reporting. For example, refs [8,10,11,13,15,16,17,19,25] do not provide model parameters (i.e., the number of parameters in the respective architectures). Regarding the PhysioNet/CinC Challenge 2016 dataset, previous works such as [19,22] reported accuracy performances of 88% and 90%, respectively. Singh et al. [22] proposed the AlexNet architecture, which achieved a 90% accuracy rate, but with an extremely high parameter count of 58 M. Chen et al. [28] proposed a CNN model combining STFT and Log-MelSpectrogram, which achieved an accuracy of 91.74%—approximately 1% higher than our model. However, their architecture required 17.5 M parameters and 416.9 M FLOPs. According to Table 23, models reporting higher accuracy on the noisier PhysioNet/CinC Challenge 2016 dataset, such as [28], adopt filtering and denoising strategies including Butterworth and Savitzky–Golay filters. In contrast, the model proposed in this study achieved 90.74% accuracy without the use of filters, relying solely on MFCC feature extraction and zero-padding segmentation, while requiring only 1.8% of the parameters and 1% of the computational cost compared to [28].

When comparing the performance of various studies on the GitHub PCG database, the models developed in [8,10,15,16,17], which employ machine learning methods, achieved high classification accuracy (99.4%, 97.9%, 99.5%, 95.13%, and 98.53%, respectively); however, the proposed WCNN-based model outperformed these models. Baghel et al. [24] used data augmentation techniques to increase the quantity of training data for a CNN model and then employed this model to classify signals from the GitHub PCG database. They achieved an accuracy of 98.6% with only 0.28 M parameters. Although the model developed in [24] uses 11% fewer parameters than that developed in this work, the model developed in [24] has 98% higher FLOPs and a lower accuracy. The WaveNet model proposed in [25] achieved an overall accuracy of 97% for detecting HVDs in PCG signals. In [12], MFCC was used for feature extraction, and a 1D CNN–LSTM model was constructed for heart sound recognition. This model outperformed that proposed in [25], achieving an accuracy of 99.4% with 0.606 M parameters. Moreover, the authors of [21] developed the Cardi-Net model for the automatic identification of heart diseases, achieving an accuracy of 98.879% with 9.5 M parameters. The numbers of parameters and FLOPs for this model are 96.7% and 97.6% higher, respectively, than those for the proposed WCNN-based model. In addition, Nguyen et al. [29] introduced a CNN-based model utilizing Log-MelSpectrogram features, achieving an accuracy of 99.337% on the GitHub PCG dataset using a 1.5-second signal segment. Since this duration is close to the 1.4 second input length used in the present work, the result serves as a meaningful reference for comparison. However, this performance came at the cost of significantly increased model complexity, requiring 1.62 M parameters and over 22.5 M FLOPs—more than five times the size and computational cost of the proposed model. Similarly, Choudhary et al. [13] proposed a CNN–GRU hybrid architecture and achieved 99.3% accuracy. While the results are promising in terms of classification accuracy, the study did not report the model size or FLOPs, making it difficult to assess its feasibility for deployment on resource-constrained devices.

Two independent models were trained and evaluated separately on the GitHub PCG and PhysioNet/CinC Challenge 2016 databases. These two datasets differ substantially in recording quality, with the former containing relatively clean signals and the latter exhibiting significant noise interference. Consequently, the comparative results between the two datasets demonstrate the proposed model’s robustness under varying noise conditions. To address this problem, paper [26]’s, paper [23]’s and paper [30]’s have evaluated their models on multiple databases. Shuvo et al. [26] developed a bidirectional-LSTM-based CardioXNet architecture and evaluated it on the GitHub PCG and PhysioNet/CinC Challenge 2016 databases, achieving accuracy values of 99.6% and 86.57%, respectively, with 0.67 M parameters. Similarly, Karhade et al. [23] used time–frequency images as input for a deep CNN model, employing 1.2 M parameters to achieve accuracy values of 99.48% and 85.16% on the aforementioned two databases, respectively. Compared with the model proposed in [23], the WCNN-based model developed in the present study achieved higher accuracy on the GitHub PCG and PhysioNet/CinC Challenge 2016 databases while using 74.9% fewer parameters and 99.3% fewer FLOPs. Wang et al. [30] proposed PCTMF-Net, combining parallel CNNs, transformer modules, and second-order spectral analysis. It achieved 99.36% accuracy on GitHub PCG and 93% on PhysioNet/CinC Challenge 2016 database. On the noisier PhysioNet/CinC 2016 dataset, our model reached 90.74%, about 2.26% lower than [30]. According to Table 23, their method includes both digital Butterworth filtering and second-order spectral analysis, which likely enhanced noise suppression. However, their model achieved only 99.36% accuracy on the cleaner GitHub PCG dataset, while the proposed model achieved 99.6%, indicating that excessive filtering may reduce discriminative feature preservation in low-noise settings. Notably, our model achieved strong performance without such filtering, showing better robustness under practical conditions. In terms of complexity, PCTMF-Net uses 2.56 M parameters, over eight times that of our WCNN-based model, highlighting its superior compactness and efficiency. This performance contrast highlights the robustness of the proposed approach, which maintains high accuracy across both clean and noisy datasets while employing a simpler preprocessing pipeline—MFCC feature extraction and zero-padding segmentation—without explicit denoising. The model achieved this efficiency using only 312,357 parameters and 4.47 M FLOPs. Furthermore, according to Table 23, several studies, such as [19,21], employed a 90%/10% training/testing split, consistent with the evaluation procedure in this work. Among them, the proposed model attained the highest accuracy on the GitHub dataset (99.6%). This consistency further validates the model’s competitive performance under standardized experimental conditions.

Table 22 presents the added f1-scores to provide a fairer comparison of model performance. However, not all referenced studies reported f1-scores; specifically, papers [14,15,18,20,22,24,25,27] did not include these values. For studies that reported precision together with recall or provided a confusion matrix (e.g., [20,22,25]), we computed the corresponding f1-scores using Equation (7), as presented in Table 22. It is worth noting that the issue of determining which class is treated as the positive class arises in binary classification. In this case, the f1-score can vary considerably depending on whether the majority or minority class is considered positive. For example, in our experiments using the PhysioNet/CinC Challenge 2016 database, when the majority (normal) class was treated as positive, the precision and recall were 93.99% and 96.48%, respectively, yielding an f1-score of 95.24%. Conversely, when the minority (abnormal) class was treated as positive, the precision and recall were 77.05% and 85.45%, resulting in an f1-score of 81.25%.

In this study, we report the f1-score based on the minority (abnormal) class, with an average f1-score of 84.94% obtained from 10-fold cross-validation. Since most previous works did not specify which class was considered positive, we calculated their f1-scores from their published confusion matrices under the same assumption (i.e., the abnormal class as positive) to ensure a fair comparison. The blue entries in Table 22 indicate the f1-scores we derived from the corresponding confusion matrices using this consistent evaluation criterion. For multi-class classification, such as the GitHub PCG database, each class is treated as the positive class in turn, and the final f1-score is obtained by averaging across all classes (macro-averaging). Based on the results in Table 22, our model achieved an f1-score of 99.5% on the GitHub PCG database and 84.94% on the PhysioNet/CinC Challenge 2016 database. Compared with other studies using the same datasets, our proposed model demonstrates comparable or superior performance on both datasets. Specifically, on the GitHub PCG database, our model’s f1-score is similar to those of Khan et al. [17] (99.4%) and Yaseen et al. [10] (99.7%), while achieving these results with significantly fewer parameters. On the PhysioNet/CinC Challenge 2016 database, our model outperforms most previous approaches such as Shuvo et al. [26] (63.29%), Singh et al. [22] (78.68%), and Wang et al. [30] (81.83%), highlighting its superior robustness to noise and data variability. In summary, Table 22 and Table 23 jointly support a detailed cross-study evaluation, illustrating that the proposed model achieves strong accuracy with minimal resource usage, while maintaining robust performance across datasets with differing levels of noise and class balance.

### 4.5. Analysis of Signal Quality and Misclassification Patterns

**Figure 15:**
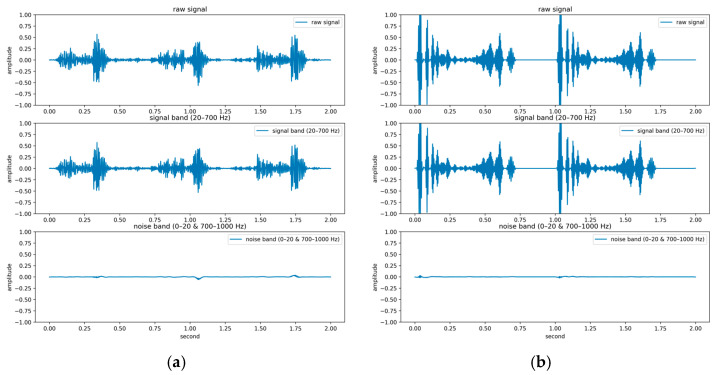
Time-domain PCG signals with different BER values from the GitHub PCG dataset: (**a**) Sample with lower BER (228.45); (**b**) Sample with higher BER (2442.15).

**Figure 16:**
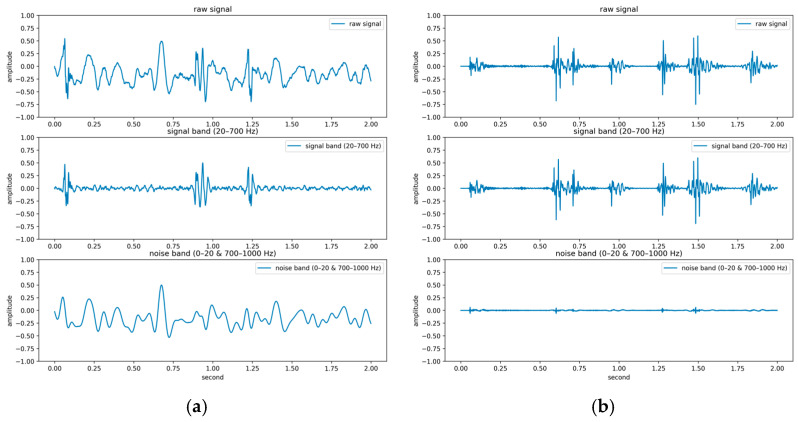
Time-domain PCG signals with different BER values from the PhysioNet/CinC Challenge 2016 dataset (excluding samples labeled as “unsure”): (**a**) Sample with lower BER (0.0878); (**b**) Sample with higher BER (128.7231).

**Figure 17:**
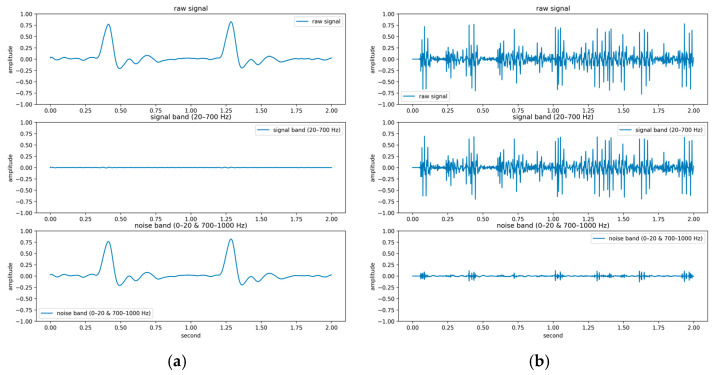
Time-domain PCG signals with different BER values from the PhysioNet/CinC Challenge 2016 dataset (samples labeled as “unsure”): (**a**) Sample with lower BER (0.0001); (**b**) Sample with higher BER (66.3786).

To systematically investigate the impact of noise on feature extraction and classification performance, a quantitative signal quality analysis was conducted. To ensure consistency in frequency resolution, all PCG recordings were resampled to 2000 Hz before frequency analysis. Based on the proposed analysis method, the GitHub PCG dataset showed a high average BER of 707.07, indicating clean and consistent signal quality. In contrast, the PhysioNet/CinC Challenge 2016 dataset exhibited a much lower average BER of 24.96, confirming greater noise contamination. Further analysis revealed that samples not labeled as “unsure” had a slightly higher average BER of 27.85, while the “unsure”-labeled samples showed an extremely low average BER of 2.06, suggesting minimal heart sound energy and severe noise interference. To further validate the correlation between the BER and signal quality, representative samples with varying BER values from both datasets were selected for visual analysis. As shown in Figure 15, Figure 16 and Figure 17, each figure consists of three rows, where the top row presents the raw time-domain signal, the middle row displays the signal filtered within the target frequency band (20–700 Hz), and the bottom row illustrates the noise-band components (0–20 Hz and 700–1000 Hz), which are considered non-informative for heart sound analysis.

Figure 15 presents two samples from the GitHub PCG dataset, with BER values of 228.45 and 2442.15 in Figure 15a and Figure 15b, respectively. Both samples exhibit clear periodic patterns and distinct S1/S2 heart sound structures. Even the sample with the lower BER (228.45) still represents a high-quality and easily identifiable signal. Figure 16 shows two samples from the PhysioNet/CinC Challenge 2016 database that are not labeled as “unsure.” The sample in Figure 16a has a BER of 0.0878, and its time-domain waveform and frequency spectrum reveal no recognizable heart sound features, reflecting a highly noisy signal. In contrast, Figure 16b (BER = 128.72) displays clear periodicity and concentrated energy in the relevant frequency bands, indicating better signal quality. Figure 17 illustrates two samples labeled as “unsure” from the PhysioNet/CinC Challenge 2016 database. The sample in Figure 17a has a BER of 0.0001 and shows virtually no observable heart sound structure, qualifying it as an extreme noise-dominated and unclassifiable example. Although the sample in Figure 17b has a slightly higher BER of 66.37, and exhibits some concentrated frequency energy, it still lacks sufficient periodic structure and remains unclassifiable.

Beyond visual inspection, further analysis was conducted to investigate how signal quality, as measured by the BER, relates to actual model performance, particularly in terms of classification errors. Classification performance across different categories was examined through confusion matrix analysis and error-type statistics. In the multi-class classification task (AS, MR, MS, MVP, N), most samples were correctly classified, with true positive counts generally reaching 19 or 20 per class (95–100%). Misclassifications—whether false positives or false negatives—were rare and typically limited to isolated instances, indicating that the model performs robustly in classes with well-defined structures and consistent features.

In the binary classification task (Normal vs. Abnormal), the confusion matrix showed strong recognition for Normal cases (TP = 219, 96.5%), while Abnormal cases had a slightly higher number of false negatives (FN = 14, 22.9%), suggesting that certain abnormal heart sounds were incorrectly identified as normal. To further investigate these errors, BER values were analyzed in relation to classification results. Among samples not labeled as “unsure” in the PhysioNet/CinC Challenge 2016 database, the average BER was 27.85. However, misclassified samples had a notably lower average BER of 19.72, compared to 27.91 for correctly classified samples. This indicates that misclassified signals were generally of lower quality, with greater noise interference and weaker heart sound structures. These findings are consistent with the known characteristics of the dataset and emphasize that environmental noise not only degrades signal quality but also significantly impacts the model’s ability to detect abnormal heart sounds, making it a key factor contributing to classification errors.

The proposed BER method effectively evaluates the signal-to-noise ratio of PCG recordings. As shown in Figure 15, Figure 16 and Figure 17, the GitHub PCG dataset exhibits cleaner signals with higher BER values, whereas the PhysioNet/CinC Challenge 2016 dataset contains more noise and thus lower BERs. Even without applying noise reduction preprocessing, the proposed model achieved 90.74% accuracy on the noisier PhysioNet dataset, demonstrating strong noise resilience. Overall, these results confirm that signal quality—quantified by BER—has a decisive impact on feature extraction and classification accuracy. The performance gap between the two datasets mainly stems from differences in data quality, validating BER as a reliable indicator of signal integrity and model robustness.

### 4.6. Results Obtained on Raspberry Pi

**Figure 18:**
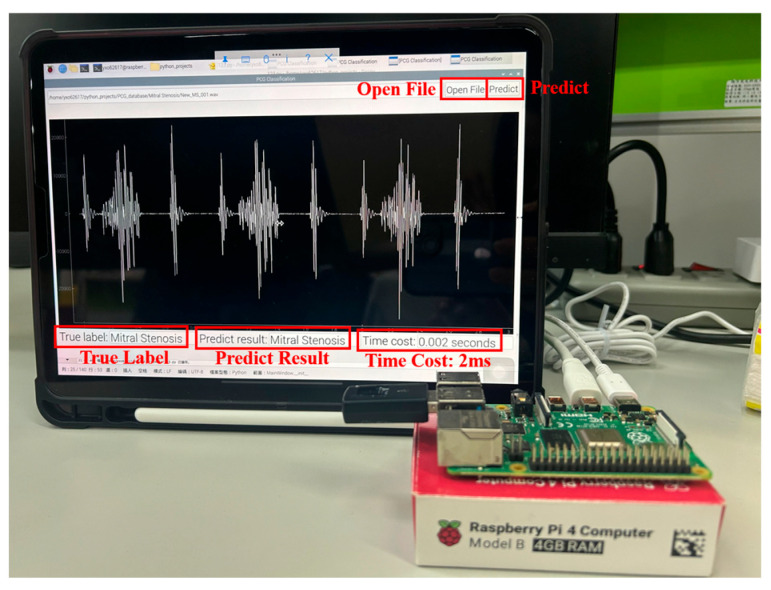
User interface of the proposed HVDs recognition system on Raspberry Pi.

The proposed WCNN-based model was implemented in Python by using Raspberry Pi 4, which has a Linux operating system. Figure 18 shows the user interface of Raspberry Pi, which had two buttons: “Open File” and “Predict.” After a .wav file was selected by clicking the “Open File” button, the file path and waveform were displayed, and the waveform could be zoomed into or zoomed out from. The true label is also displayed. Clicking the “Predict” button resulted in the generation of the predicted result, which was displayed with the prediction time.

The real-time analysis process includes the pre-processing stage for input data, the computational time for model inference, and the time required for the output of result. As presented in Table 24, the corresponding pseudocode summarizes this process. The procedure begins with input data preparation, followed by preprocessing, model execution, and extraction of the classification result. A timing mechanism records both the start and end times, allowing the computation of the overall time cost. This methodology accurately reflects the classification time cost of the model in practical operation, as it integrates all essential stages including preprocessing, computational inference, and output generation. Based on 20 test samples, the average classification time cost was measured at 1.87 ms. As displayed in Figure 18, the proposed model achieved real-time prediction on Raspberry Pi, with its processing time being 2 ms during one test.

### 4.7. Future Work

Wearable devices have shown significant advantages in HVDs detection, such as portability, continuous monitoring, and user-friendly characteristics. These features enable non-invasive, real-time data collection during daily activities, making them especially suitable for populations in remote or resource-limited areas. However, the limited computational power and battery life of these devices pose challenges to the complexity of algorithms and real-time feedback capabilities. Moreover, noise and motion artifacts can degrade signal quality, further affecting detection accuracy. To address these challenges, this study designed a lightweight model that leverages MFCCs to extract effective heart sound features, combined with a KWC layer to enhance the recognition of key features, ensuring stable performance under the resource constraints of wearable hardware. Despite the proposed model’s strong accuracy and practicality, it is crucial to further investigate its applicability and potential weaknesses in diverse environments. In addition to these challenges, our analysis also revealed that samples with lower BER tended to exhibit higher misclassification rates due to weaker heart sound structures and increased noise. Future work may explore the integration of BER-based quality indicators into signal selection or confidence estimation frameworks, especially for real-time applications.

The proposed model’s recognition accuracy may decrease in the presence of noise. To address this issue, future studies could focus on improving signal preprocessing and digital filtering techniques, such as using spectral filtering, wavelet transforms, and adaptive filters to effectively eliminate environmental noise and high-frequency interference. Additionally, combining GradCAM technology to focus on key heart sound segments unaffected by interference could enhance recognition accuracy. Furthermore, end-to-end models that integrate filtering processes with the proposed classification models, along with adversarial training and noise data augmentation strategies, could strengthen the proposed model’s robustness and generalization capabilities under various environmental noises. These improvements will further enhance the proposed model’s stability and practicality, providing stronger support for future clinical applications.

## 5. Conclusions

This study presents an integrated and deployable system for HVD recognition based on PCG signals, where the proposed WCNN-based architecture is one component within a broader and carefully designed workflow. In addition to introducing a lightweight and noise-resilient classifier, the system incorporates MFCC-based feature optimization, signal quality assessment through BER, and balanced evaluation across both clean and noisy datasets. The results show the proposed model exhibited the highest accuracy on the GitHub PCG and PhysioNet/CinC Challenge 2016 databases (99.6% and 90.74%, respectively) with a smaller number of parameters and FLOPs (approximately 312.357 K and 4.5 M, respectively). The successful implementation on a Raspberry Pi confirms the system’s suitability for real-time use in wearable or embedded environments. Overall, this work demonstrates not only high classification performance but also a practical and generalizable system design for cardiovascular screening in real-world applications.

In summary, this research makes five crucial contributions to the literature. First, through the strategic selection of MFCC feature dimensions, the number of model parameters was considerably reduced without compromising model accuracy. Second, the use of a KWC layer in the proposed model enhanced its attention to crucial audio features, thereby optimizing its performance. Third, the comprehensive evaluation strategy, which involved conducting 10-fold cross-validation on two datasets, enabled robust assessment of the proposed model’s accuracy. Fourth, the integration and validation of the BER metric provided a practical framework for quantifying signal quality and analyzing classification reliability under noise. Finally, the model was successfully deployed on a Raspberry Pi, demonstrating its ability to perform real-time HVD recognition under low-resource conditions. Thus, the proposed lightweight computational architecture, which uses low numbers of parameters and FLOPs, can be implemented in wearable devices. The proposed model is expected to become a valuable tool for the future clinical diagnosis of HVDs.

## Figures and Tables

**Table 1:** Pseudocode for WCNN and KWC Layer.

Line	Pseudocode
01	Input: MFCC feature map F[C×H×W]
02	for each convolutional layer:
03	F = Conv (F) -> ReLU -> BatchNorm
04	end for
05	# Compute weights using KWC layer
06	for each channel c in F:
07	$w_{c}$ = average ($F_{c}$)
08	$F_{c}^{\prime }$ = $F_{c}\times w_{c}$
09	end for
10	# Flatten and classify
11	$F_{flat}$ = Flatten ($F_{c}^{\prime }$)
12	Output: FullyConnected ($F_{flat}$) -> Softmax

**Table 2:** Information on the Layers of the Proposed WCNN Model.

Layer (Type)	No. Filter	Kernel Size × Stride	AF ^1^	BN ^2^	Dropout	Params ^3^	Output Size
Input							17 × 13 × 1
Convolution 1	32	(2 × 2) × 1	ReLU ^4^	√ ^5^		160	16 × 12 × 32
Convolution 2	64	(2 × 2) × 1	ReLU	√		8256	15 × 11 × 64
Convolution 3	64	(2 × 2) × 2	ReLU	√	√	16,448	7 × 5 × 64
KWC	− ^6^	−	−	−	−	0	1 × 1 × 64
Flatten	−	−	−	−	−	0	2240 × 1
Fully Connected	−	−	ReLU	−	√	286,848	128 × 1
Output			Softmax	−		645	5 × 1
Total parameters: 312,357							

^1^ Activation function. ^2^ Batch normalization. ^3^ Number of parameters. ^4^ Rectified linear unit. ^5^ The symbol “√” indicates the operation is applied. ^6^ The symbol “−” denotes the operation is not applied.

**Table 3:** Effects of Hyperparameter Settings for the Proposed Classification Model.

Database	Epoch	Batch Size	Learning Rate	Optimizer	Loss Function
GitHub PCG [10]	100	16	0.001	Adam	Categorical Cross Entropy
PhysioNet [33]	100	128	0.001	Adam	Binary Cross Entropy

**Table 4:** Experimental Setup.

Hardware Platform	Version
CPU	AMD Ryzen 7 5700X
GPU	NVIDIA GeForce RTX2060 SUPER
RAM	32G (DDR4)
**Software platform**	**Version**
Os	Window 10 Pro
python	3.8.18
Tensorflow	2.10.1
Raspberry Pi 4	Linux 6.1.25-v8+

**Table 5:** Summary of the PCG Databases Used in This Work.

Database	Recordings	Classes	Duration	Sample Rate (Hz)	Noise
GitHub PCG [10]	1000	5	1.2–4 s	8000	N/A
PhysioNet [33]	3240	3	5–120 s	2000	breathing, speech, stethoscope friction, and intestinal sounds

**Table 6:** Accuracy (%) of the Proposed Classification Architecture for the GitHub PCG Database Under Different Numbers of MFCC Feature Dimensions and Feature Frames.

	Frame	9	10	11	12	13	14	15	16	Average
Dim. *										
13		98.7	99	98.9	98.9	98.4	99.1	99.3	99.3	98.95
14		98.7	99	98.6	99	99.3	99.2	99.2	98.8	98.98
15		98.5	98.8	99.4	98.6	99.1	99.3	99.3	99.4	99.05
16		98.7	99	99.2	98.9	99.2	99	99.2	98.9	99.01
17		98.9	99.3	99.4	99.5	99.6	99.3	99.2	99.2	99.3
18		99	98.8	99.4	99.4	99.4	99	99.2	99	99.15
19		99.1	99.4	99.2	99.5	99.5	99.2	99.4	99	99.29
20		99.2	99.3	99.3	99.4	99.4	99.2	99	99.1	99.24
average		98.85	99.08	99.18	99.15	99.24	99.16	99.23	99.088	

* Number of MFCC feature dimensions.

**Table 7:** Accuracy (%) of the Proposed Classification Architecture for the PhysioNet/CinC Challenge 2016 Database Under Different Numbers of MFCC Feature Dimensions and Feature Frames.

	Frame	9	10	11	12	13	14	15	16	Average
Dim. *										
13		89.83	90.59	90.45	90.07	90.14	90	90.14	90.31	90.191
14		90.31	90.24	89.93	90.04	90.38	90.42	89.93	90.1	90.169
15		90.17	89.93	89.83	90.42	90.55	90.59	90.45	90.24	90.273
16		90.45	90	90.38	90.42	90.14	90.49	90.1	89.86	90.23
17		90.14	90.52	90.56	90.49	90.74	90.38	90.38	90.24	90.431
18		90.24	90.21	90.45	90.31	90.59	90.35	90.24	90.63	90.378
19		90.31	90.07	90.21	90.28	90.59	90.66	90.35	90.35	90.353
20		90.14	90.49	90.38	90.52	90.59	90.31	90.52	90.07	90.378
average		90.199	90.256	90.274	90.319	90.465	90.4	90.264	90.225	

* Number of MFCC feature dimensions.

**Table 8:** Variations in the Number of Model Parameters with the Numbers of MFCC Feature Dimensions and Feature Frames.

	Frame	9	10	11	12	13	14	15	16
Dim. *									
13		148,517	189,477	189,477	230,437	230,437	271,397	271,397	312,357
14		173,093	222,245	222,245	271,397	271,397	320,549	320,549	369,701
15		173,093	222,245	222,245	271,397	271,397	320,549	320,549	369,701
16		197,669	255,013	255,013	312,357	312,357	369,701	369,701	427,045
17		197,669	255,013	255,013	312,357	312,357	369,701	369,701	427,045
18		222,245	287,781	287,781	353,317	353,317	418,853	418,853	484,389
19		222,245	287,781	287,781	353,317	353,317	418,853	418,853	484,389
20		246,821	320,549	320,549	394,277	394,277	468,005	468,005	541,733

* Number of MFCC feature dimensions.

**Table 9:** Effects of The Number of MFCC Feature Dimensions on the Number of Parameters and Accuracy of the Proposed WCNN Model under the Same Number of Frames (13).

MFCC	Static Feature		Dynamic Feature
dimension	13	17	39
parameter	230,437	312,357	762,917
accuracy	98.4%	99.6%	99.2%

**Table 10:** Effect of Batch Size on Model Accuracy for Both Databases.

GitHub PCG Database		PhysioNet	
batch size	accuracy	batch size	accuracy
8	99.3%	64	89.62%
16	99.6%	128	90.7%
32	99.4%	256	89.45%

**Table 11:** Comparison Results of the Proposed WCNN Model and the Proposed WCNN Model without KWC Layer using the GitHub PCG Databased.

	Accuracy	Precision	Recall	f1-Score	Kappa	Parameters
The proposed WCNN model	99.6%	99.6%	99.6%	99.6%	99.5%	312,357
The proposed WCNN model without KWC layer	98.9%	98.9%	98.9%	98.9%	98.6%	312,357

**Table 12:** Comparison Results of the Proposed WCNN Model and the Proposed WCNN Model without KWC Layer using the PhysioNet/Cinc Challenge 2016 Dataset.

	Accuracy	Precision	Recall	f1-Score	Parameters
The proposed WCNN model	90.74%	85.85%	83.46%	84.94%	312,357
The proposed WCNN model without KWC layer	88.37%	76.74%	64.71%	69.87%	312,357

**Table 13:** Performance of the Proposed Classification Model in 10-fold Cross-Validation for the GitHub PCG Database.

No.	Accuracy (%)	Precision (%)	Recall (%)	f1-Score (%)	Kappa (%)
1	98	98.05	98	97.99	97.5
2	100	100	100	100	100
3	100	100	100	100	100
4	100	100	100	100	100
5	100	100	100	100	100
6	99	99.05	99	98.99	98.75
7	99	99.05	99	98.99	98.75
8	100	100	100	100	100
9	100	100	100	100	100
10	100	100	100	100	100
Avg. ± Std.	99.6 ± 0.66	99.6 ± 0.64	99.59 ± 0.66	99.61 ± 0.66	99.5 ± 0.83

**Table 14:** Average Accuracy of the Proposed Classification Model for Each Category in 10-fold Cross-Validation with the GitHub PCG Database.

Category	Aortic Stenosis	Mitral Regurgitation	Mitral Stenosis	Mitral Valve Prolapse	Normal
Accuracy	99.5%	99%	100%	99.5%	100%

**Table 15:** Performance of the Proposed Classification Model in 10-fold Cross-Validation for the PhysioNet/CinC Challenge 2016 Database.

No.	Accuracy (%)	Precision (%)	Recall (%)	f1-Score (%)
1	91.67	88.88	82.88	85.39
2	92.01	82.02	80.70	81.33
3	89.58	84.86	82.88	83.81
4	90.28	83.38	79.04	80.90
5	88.35	87.08	82.88	84.75
6	90.97	83.40	82.06	82.70
7	90.28	85.54	87.00	91.32
8	90.97	91.23	82.18	85.65
9	92.36	83.89	85.10	84.48
10	90.97	88.24	89.92	89.04
Avg. ± Std.	90.74 ± 1.19	85.85 ± 2.78	83.46 ± 2.98	84.94 ± 3.07

**Table 16:** Average Accuracy of the Proposed Classification Model for Each Category in 10-fold Cross-Validation with the PhysioNet/CINC Challenge 2016 Database.

Category	Normal	Abnormal
Accuracy	96.48%	77%

**Table 17:** AUC-ROC and AP of the Proposed Classification Model in 10-fold Cross-validation with GitHub PCG Database.

	AUC-ROC (%)	mAP (%)
0	99.93	99.70
1	100	100
2	100	100
3	99.9	99.63
4	100	100
5	100	100
6	99.99	99.95
7	99.99	99.95
8	100	100
9	100	100
average	99.98	99.92

**Table 18:** AUC-ROC and AP of the Proposed Classification Model in 10-fold Cross-validation with the PhysioNet/CinC Challenge 2016 Database.

	AUC-ROC (%)	AP (%)
0	87.74	86.45
1	84.06	84.27
2	86.1	89.45
3	83.64	85.75
4	77.68	86.57
5	85.26	84.96
6	86.25	88.81
7	86.55	83.12
8	87.35	89.88
9	83.82	88.77
average	84.85	86.8

**Table 19:** The Detailed 10-Fold Confusion Matrix of the Proposed Model under Balanced Sampling.

Predicted			AR	MR	MS	MVP	N	Predicted			AR	MR	MS	MVP	N
**Actual**	Fold-1	AR	20	0	0	0	0	**Actual**	Fold-6	AR	19	1	0	0	0
		MR	0	19	1	0	0			MR	0	20	0	0	0
		MS	0	0	20	0	0			MS	0	0	20	0	0
		MVP	1	0	0	19	0			MVP	0	0	0	20	0
		N	0	0	0	0	20			N	0	0	0	0	20
	Fold-2	AR	20	0	0	0	0		Fold-7	AR	20	0	0	0	0
		MR	0	20	0	0	0			MR	0	19	1	0	0
		MS	0	0	20	0	0			MS	0	0	20	0	0
		MVP	0	0	0	20	0			MVP	0	0	0	20	0
		N	0	0	0	0	20			N	0	0	0	0	20
	Fold-3	AR	20	0	0	0	0		Fold-8	AR	20	0	0	0	0
		MR	0	20	0	0	0			MR	0	20	0	0	0
		MS	0	0	20	0	0			MS	0	0	20	0	0
		MVP	0	0	0	20	0			MVP	0	0	0	20	0
		N	0	0	0	0	20			N	0	0	0	0	20
	Fold-4	AR	20	0	0	0	0		Fold-9	AR	20	0	0	0	0
		MR	0	20	0	0	0			MR	0	20	0	0	0
		MS	0	0	20	0	0			MS	0	0	20	0	0
		MVP	0	0	0	20	0			MVP	0	0	0	20	0
		N	0	0	0	0	20			N	0	0	0	0	20
	Fold-5	AR	20	0	0	0	0		Fold-10	AR	20	0	0	0	0
		MR	0	20	0	0	0			MR	0	20	0	0	0
		MS	0	0	20	0	0			MS	0	0	20	0	0
		MVP	0	0	0	20	0			MVP	0	0	0	20	0
		N	0	0	0	0	20			N	0	0	0	0	20

**Table 20:** The Detailed 10-Fold Confusion Matrix of the Proposed Model under Random Sampling.

Predicted			AR	MR	MS	MVP	N	Predicted			AR	MR	MS	MVP	N
**Actual**	Fold-1	AR	19	0	0	0	0	**Actual**	Fold-6	AR	14	0	0	0	0
		MR	0	22	0	0	0			MR	0	17	0	0	0
		MS	0	0	26	0	0			MS	0	0	17	0	0
		MVP	0	0	0	15	0			MVP	0	0	0	22	0
		N	0	0	0	0	18			N	0	0	0	0	30
	Fold-2	AR	20	0	0	0	0		Fold-7	AR	16	1	0	0	0
		MR	0	19	0	0	0			MR	0	22	0	0	0
		MS	0	0	14	0	0			MS	0	0	22	0	0
		MVP	0	1	0	27	0			MVP	0	0	0	23	0
		N	0	0	0	0	19			N	0	0	0	0	16
	Fold-3	AR	21	0	0	0	0		Fold-8	AR	21	0	0	0	0
		MR	0	19	0	0	0			MR	0	14	0	0	0
		MS	0	0	20	0	0			MS	0	0	21	0	0
		MVP	2	0	0	19	0			MVP	0	0	0	18	0
		N	0	0	0	0	19			N	0	0	0	0	26
	Fold-4	AR	21	0	0	0	0		Fold-9	AR	24	0	0	0	0
		MR	0	16	0	0	0			MR	0	21	0	0	0
		MS	0	0	18	0	0			MS	0	0	20	0	0
		MVP	0	1	0	27	0			MVP	0	0	0	19	0
		N	0	0	0	0	17			N	0	0	0	0	16
	Fold-5	AR	16	0	0	0	0		Fold-10	AR	27	0	0	0	0
		MR	0	24	0	0	0			MR	0	25	1	0	0
		MS	0	0	25	0	0			MS	0	0	17	0	0
		MVP	0	0	0	13	0			MVP	0	0	0	13	0
		N	0	0	0	0	22			N	0	0	0	0	17

**Table 21:** Average Accuracy of the Proposed Classification Model for Each Category under 10-Fold Cross-Validation on the GitHub PCG Database with Random Sampling.

Category	Aortic Stenosis	Mitral Regurgitation	Mitral Stenosis	Mitral Valve Prolapse	Normal
Accuracy	99.4%	99.6%	100%	98.3%	100%

**Table 22:** Comparison Between the Proposed WCNN-Based Model and Other Models from the Literature.

Method	Years	Parameters	FLOPs	Classifier	Database	Accuracy (%)	f1-Score (%)
Tang et al. [19]	2018	--	--	SVM	PhysioNet	88	--
Yaseen et al. [10]	2018	--	--	SVM	GitHub PCG	97.9	99.7
Ghosh et al. [16]	2019	--	--	RF	GitHub PCG	95.13	--
Singh et al. [22]	2019	58,289,538	2,262,336,576	AlexNet	PhysioNet	90	78.68
Baghel et al. [24]	2020	277,861	225,602,432	CNN	GitHub PCG	98.6	98.5
Oh et al. [25]	2020	--	--	WaveNet	GitHub PCG	97	--
Ail Kobat et al. [15]	2021	--	--	KNN	GitHub PCG	99.5	--
Shuvo et al. [26]	2021	670,000	--	CardioXNet	GitHub PCG	99.6	99.4
					PhysioNet	86.57	63.29
Netto et al. [12]	2021	606,949	--	1D CNN-LSTM	GitHub PCG	99.4	98.8
Khan et al. [17]	2022	--	--	RF + multiboost	GitHub PCG	98.53	96.4
Arslan et al. [11]	2022	--	--	DNN	GitHub PCG	98.8	98.8
Khan et al. [21]	2022	9,514,889	183,823,360	Cardi-Net	GitHub PCG	98.879	97.13
Karhade et al. [23]	2022	1,249,480	596,714,752	deep CNN	GitHub PCG	99.48	--
					PhysioNet	85.16	85.16
Chen et al. [28]	2023	17,477,682	416,940,544	CNN	PhysioNet	91.74	--
Nguyen et al. [29]	2023	1,623,901	22,577,640	CNN	GitHub PCG	99.337	99.332
Wang et al. [30]	2023	2,561,413	--	PCTMF-Net	GitHub PCG	99.36	99.3
					PhysioNet	93	81.83
Choudhary et al. [13]	2024	9,058,389	--	CNN-GRU	GitHub PCG	99.3	99.3
Kobat et al. [8]	2024	--	--	SVM	GitHub PCG	99.4	99.39
This work		312,357	4,474,112	Weighted CNN	GitHub PCG	99.6	99.5
					PhysioNet	90.74	84.94

Red text indicates the results of the proposed model. Blue text represents the F1-score calculated using the confusion matrix provided in the referenced paper, where the abnormal class is considered the positive class.

**Table 23:** Data Pre-processing and Distribution Between the Proposed WCNN-Based Model and Other Models from the Literature.

Method	Pre-Processing	Distribution Data (Training, Testing, Validation)
Tang et al. [19]	Highpass filter, Spike removal, Normalization, Segmentation	90%, 10%
Yaseen et al. [10]	MFCC, DWT	80%, 20%
Ghosh et al. [16]	Bandpass filter, Segmentation, WST	70%, 30%
Singh et al. [22]	Bandpass filter, Spike removal, CWT	50%, 50%
Baghel et al. [24]	Background deformation, Normalization, Segmentation, Gaussian Butterworth bandpass filter	--
Oh et al. [25]	Normalization, Segmentation with zero-padding	85.5%, 10%, 4.5%
Ail Kobat et al. [15]	IBP, NCA	--
Shuvo et al. [26]	Lowpass filter, Normalization, Segmentation with zero-padding	70%, 20%, 10%
Netto et al. [12]	Excluded noise/low-amplitude recordings, Normalization, MFCC	60%, 25%, 15%
Khan et al. [17]	Butterworth bandpass filter, Segmentation with Shannon energy envelope, Normalization, Zero-padding	--
Arslan et al. [11]	HHT with EMD, MFCC	80%, 20%
Khan et al. [21]	Bandpass filter, Segmentation with zero-padding, Normalization	90%, 10%
Karhade et al. [23]	Gaussian Butterworth bandpass filter, Normalization, Segmentation with zero-padding, CWT	78.75%, 10%, 11.25%
Chen et al. [28]	Resampling, Butterworth and Savitzky–Golay filter, STFT-based log-Mel spectrum	--
Nguyen et al. [29]	Segmentation, DFT-based log-Mel spectrum, Zero-center normalization	70%, 30%
Wang et al. [30]	Butterworth bandpass filter, Downsampling, Normalization, Segmentation with Overlap, Second-order spectral analysis	60%, 20%, 20%
Choudhary et al. [13]	Downsampling, Highpass Butterworth filter, Segmentation with zero-padding, Z-score Normalization, DWT based on Coiflet-5	70%, 30%
Kobat et al. [8]	Bandpass filter, Normalization, Segmentation with zero-padding, STFT + Bicubic, Shannon energy envelope	--
This work	Segmentation with zero-padding, MFCC	90%, 10%

**Table 24:** Pseudocode for Real-Time Time Cost Analysis.

Line	Pseudocode
01	Input: raw input data x_raw
02	start_time = current_time()
03	x_pre = Preprocess(x_raw)
04	predict_result = ModelInference(x_pre)
05	end_time = current_time()
06	time_cost = end_time–start_time
07	Output: predict_result, time_cost

## Data Availability

The PCG datasets used in this study are publicly available. The GitHub PCG database is available in GitHub at https://github.com/yaseen21khan/Classification-of-Heart-Sound-Signal-Using-Multiple-Features- (accessed on 21 October 2025), reference number [10]. The PhysioNet/CinC Challenge 2016 can be accessed from PhysioNet at https://physionet.org. Additional experimental results are available from the corresponding author upon reasonable request.
